# Tumor-educated Gr1^+^CD11b^+^ cells drive breast cancer metastasis via OSM/IL-6/JAK–induced cancer cell plasticity

**DOI:** 10.1172/JCI166847

**Published:** 2024-01-18

**Authors:** Sanam Peyvandi, Manon Bulliard, Alev Yilmaz, Annamaria Kauzlaric, Rachel Marcone, Lisa Haerri, Oriana Coquoz, Yu-Ting Huang, Nathalie Duffey, Laetitia Gafner, Girieca Lorusso, Nadine Fournier, Qiang Lan, Curzio Rüegg

**Affiliations:** 1 Pathology Unit, Department of Oncology, Microbiology and Immunology (OMI), Faculty of Science and Medicine, University of Fribourg, Fribourg, Switzerland.; 2 Translational Data Science Group, Swiss Institute of Bioinformatics, Lausanne, Switzerland.; 3 Cell and Tissue Dynamics Research Program, Institute of Biotechnology, Helsinki Institute of Life Science (HiLIFE), University of Helsinki, Helsinki, Finland.

**Keywords:** Oncology, Breast cancer

## Abstract

Cancer cell plasticity contributes to therapy resistance and metastasis, which represent the main causes of cancer-related death, including in breast cancer. The tumor microenvironment drives cancer cell plasticity and metastasis, and unraveling the underlying cues may provide novel strategies for managing metastatic disease. Using breast cancer experimental models and transcriptomic analyses, we show that stem cell antigen-1 positive (SCA1^+^) murine breast cancer cells enriched during tumor progression and metastasis had higher in vitro cancer stem cell–like properties, enhanced in vivo metastatic ability, and generated tumors rich in Gr1^hi^Ly6G^+^CD11b^+^ cells. In turn, tumor-educated Gr1^+^CD11b^+^ (Tu-Gr1^+^CD11b^+^) cells rapidly and transiently converted low metastatic SCA1^–^ cells into highly metastatic SCA1^+^ cells via secreted oncostatin M (OSM) and IL-6. JAK inhibition prevented OSM/IL-6–induced SCA1^+^ population enrichment, while OSM/IL-6 depletion suppressed Tu-Gr1^+^CD11b^+^–induced SCA1^+^ population enrichment in vitro and metastasis in vivo. Moreover, chemotherapy-selected highly metastatic 4T1 cells maintained high SCA1^+^ positivity through autocrine IL-6 production, and in vitro JAK inhibition blunted SCA1 positivity and metastatic capacity. Importantly, Tu-Gr1^+^CD11b^+^ cells invoked a gene signature in tumor cells predicting shorter overall survival (OS), relapse-free survival (RFS), and lung metastasis in breast cancer patients. Collectively, our data identified OSM/IL-6/JAK as a clinically relevant paracrine/autocrine axis instigating breast cancer cell plasticity and triggering metastasis.

## Abstract 2

Tumor-educated Gr1^+^CD11b^+^ cells convert low metastatic SCA1^-^ breast cancer cells into highly metastatic SCA1^+^ cells via secreted OSM and IL6 and JAK signaling.

## Graphical abstract

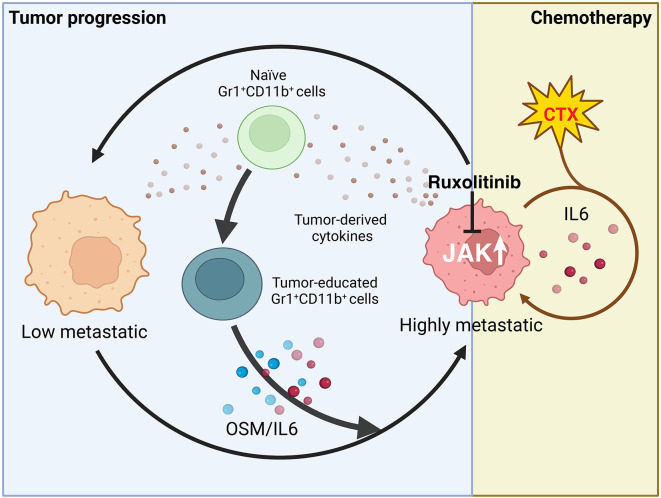

## Introduction

Metastasis accounts for over 90% of cancer-related deaths, calling for new strategies to prevent and treat metastasis (1). Recent studies using single-cell lineage tracing and single-cell RNA-Seq (scRNA-Seq) technologies have provided detailed information about intratumor heterogeneity (2, 3), whereby genetically, epigenetically, and functionally diverse subpopulations of cancer cells exist within the tumor, spatially and temporally (4). Intratumor heterogeneity may arise by modulating cancer cell plasticity, especially of cancer stem cells (CSCs), through cell-intrinsic and -extrinsic mechanisms (5, 6).

Multiple CSC subpopulations appear to coexist within the tumor mass, resulting in a high degree of tumor cell heterogeneity, including in breast cancer (4, 7–10). CSCs can acquire metastasis-initiating capacities (11) and resistance to therapy, resulting in increased aggressiveness (12) and relapse (4). Moreover, non-CSCs within the tumor bulk may acquire CSC properties to repopulate the tumor (4). While CSCs are generally defined by their ability to initiate tumors and metastasis in low numbers in vivo, several CSC-associated surface markers have been reported, including CD44, CD24, stem cell antigen-1 (SCA1), CD61, and CD49f, and used to phenotypically identify breast CSCs (13, 14).

The tumor microenvironment (TME) can promote tumor progression and metastasis through multiple mechanisms, including promotion of angiogenesis, cell survival, invasion, epithelial-mesenchymal transition (EMT), and immunosuppression (15–20). Recently, TME was reported as instigating tumor cell plasticity and tumor heterogeneity (21, 22), including the expansion of CSCs with metastatic ability (also known as metastasis-initiating cells) in different cancers (15, 23, 24). Accurate characterization of the regulation of tumor plasticity and heterogeneity by the TME may reveal novel opportunities for developing effective antimetastatic therapies (7, 25).

TME-derived oncostatin M (OSM) was shown to mediate tumor progression and CSC expansion by activating its receptor, OSMR (26). OSM belongs to the IL-6 family of cytokines (including IL-6, IL-11, and LIF) (27, 28), whose members bind to dimeric receptors sharing a common subunit (gp130 or IL-6ST) and activate JAK/STAT, RAS/MAPK, and PI3K/AKT pathways (29, 30). Increased OSM or IL-6 expression correlates with reduced survival in breast cancer patients (31, 32). OSM was shown to drive breast cancer progression and metastasis through direct effects on cancer cells, such as suppression of estrogen receptor (ER) expression (32) and promotion of EMT (26, 33), and indirect effects via TME cells, in particular the reprogramming of tumor-associated macrophages and fibroblasts (34–37).

Here, by assessing the metastatic evolution of murine breast cancer models in silico and in vivo, we observed that the SCA1^+^ tumor cell subpopulation is enriched during tumor progression. We show that tumor-educated Gr1^+^CD11b^+^ (Tu-Gr1^+^CD11b^+^) cells, rather than Gr1^+^CD11b^+^ cells from spleen (Spl-Gr1^+^CD11b^+^) or bone marrow (BM-Gr1^+^CD11b^+^) from tumor-bearing mice, effectively modulate tumor plasticity via OSM/IL-6/JAK signaling by rapidly and transiently converting SCA1^–^ cells into SCA1^+^ cells with high metastatic capacity. Chemotherapy-resistant 4T1 cells were enriched for metastatic SCA1^+^ cells via a persistent autocrine IL-6/JAK signaling loop. A short in vitro treatment of chemoresistant cells with the JAK inhibitor ruxolitinib suppressed their in vivo metastatic capacity. Importantly, Tu-Gr1^+^CD11b^+^ invoked a gene expression signature in 4T1 cells that predicted lung metastasis, shorter overall survival (OS), and relapse-free survival (RFS) in breast cancer patients, emphasizing the clinical relevance of these findings.

## Results

### A SCA1^+^ tumor cell population is enriched during tumor progression and has higher in vivo metastatic capacity.

**Figure 1:**
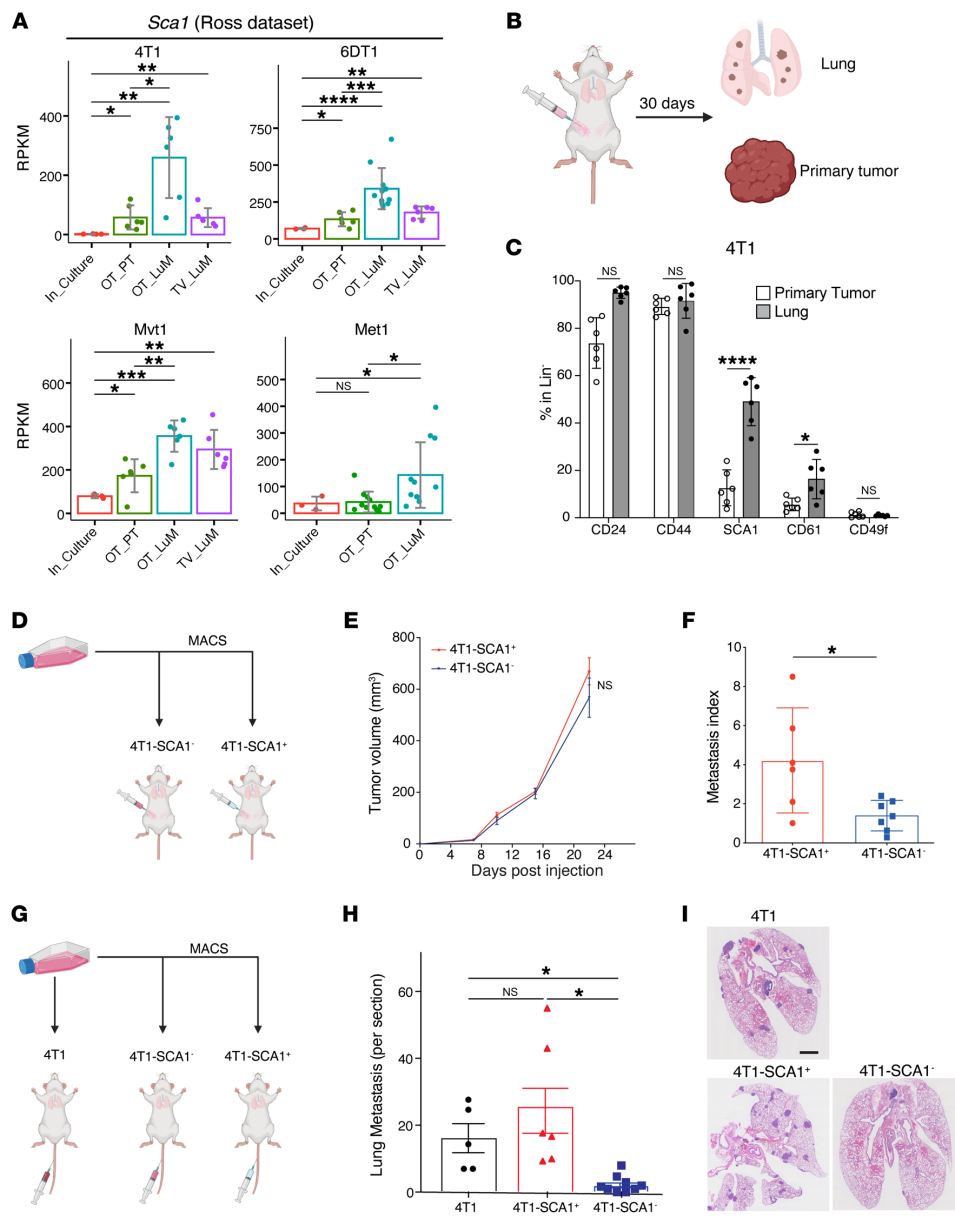
SCA1^+^ population is enriched during in vivo metastasis across multiple breast cancer models. (**A**) *Sca1* mRNA expression in the metastatic murine breast cancer models 4T1, 6DT1, Mvt1, and Met1, extracted from the Ross data set (39). Analyzed samples consist of cultured cells (In_Culture), orthotopic injected PTs (OP_PT), spontaneous lung metastases (OP_LuM), and lung metastases induced by i.v. injection (TV_LuM). Data are represented as the mean of reads per kilobase of transcript per million mapped reads (RPKM) ± SD. (**B**) Experimental setup for in vivo experimental validation. 4T1 tumor cells were orthotopically injected into the fourth mammary fat pad. Thirty days later, cells from PTs and lungs were isolated to examine CSC marker expression by flow cytometry. (**C**) Frequency of CSC marker expression in PTs and lung metastases. Results are shown as percentages of CD24-, CD44-, SCA1-, CD61-, and CD49f-positive cells gated in lineage-negative cells (CD45^–^CD31^–^TER119^–^). *n* = 6/group. (**D**–**F**) Experimental setup (**D**) of the in vivo experiment to assess tumor growth (**E**) and lung metastatic ability (metastatic index) (**F**) of 4T1-SCA1^+^ and 4T1-SCA1^–^ populations isolated from tumors induced by parental 4T1 cells orthotopically injected into the fourth mammary fat pads. Metastases are assessed 21 days after tumor cell injection. *n* = 8/group. (**G**–**I**) Experimental setup (**G**) of in vivo experiment to assess lung colonization capacity upon tail-vein injection of sorted parental 4T1, 4T1-SCA1^+^, and 4T1-SCA1^–^ cells. Lung metastatic nodule numbers (**H**) and representative images (**I**) of lungs from mice 10 days after injection. *n* = 5–6/group. Scale bar: 1 mm. Data are represented as means ± SEM and are representative of 3 independent experiments for **C**, **E**, **F**, and **H**. *P* values were calculated using unpaired, 2-tailed Student’s *t* test with Holm’s correction (**A**); unpaired 2-tailed Student’s *t* test (**C** and **F**); 2-way ANOVA with Tukey’s multiple-comparison test (**E**); or 1-way ANOVA with Tukey’s multiple-comparison test (**H**). *P < 0.05; **P < 0.01; ***P < 0.001; ****P < 0.0001.

To investigate tumor cell heterogeneity during tumor progression, we first examined the expression of reported breast CSC markers *Cd24*, *Cd44*, *Cd61*, *Sca1*, and *Cd49f* (38) in the RNA-Seq data set from Ross et al. (39) encompassing several murine breast cancer models. This data set includes data from cultured cancer cells (In_Culture), orthotopic primary tumors (OT_PT), spontaneous lung metastases (OT_LuM), and experimental lung metastases (i.e., upon tail-vein injection, TV_LuM) (Figure 1A and Supplemental Figure 1A; supplemental material available online with this article; https://doi.org/10.1172/JCI166847DS1). *Sca1* expression was consistently elevated in spontaneous lung metastasis in 4T1, 6DT1, Mvt1, and Met1 models compared with the respective primary tumors (PTs). In 4T1, 6DT1, and Mvt1 models, *Sca1* expression was also elevated in experimental lung metastases compared with cultured cells. The expression of *Cd24*, *Cd44*, *Cd61*, and *Cd49f* was either unaltered or exhibited inconsistent patterns during progression (Supplemental Figure 1A). Thus, increased expression of *Sca1* is consistently observed during metastasis across different preclinical breast cancer models.

To investigate whether the increased *Sca1* expression within the tumor mass was due to increased gene expression in all cancer cells or to the enrichment of a SCA1^+^ population, we orthotopically injected 4T1 cells and determined the frequency of different cell populations present in the PT and lung metastases 30 days later by flow cytometry (Figure 1B). The frequency of a SCA1^+^ population, and to a lesser extent of a CD61^+^ population, increased in lung metastases compared with PTs (Figure 1C). In contrast, the CD24^+^, CD44^+^, and CD49f^+^ populations were not significantly altered. Enrichment of SCA1^+^ and CD61^+^ populations in lung metastasis was confirmed in the D2A1 breast cancer model (Supplemental Figure 1B).

These observations prompted us to ask whether SCA1^+^ cells actively contribute to metastasis formation. To this end, we isolated 4T1-SCA1^+^ and 4T1-SCA1^–^ cells by magnetic activated cell sorting (MACS) from parental 4T1 cells, which contain low frequencies of SCA1^+^ populations (10%–15%) (Supplemental Figure 1C), and examined their metastatic capacity in vivo. Orthotopically injected 4T1-SCA1^+^ cells formed significantly more lung metastases than 4T1-SCA1^–^ cells, while no significant difference was observed in PT growth (Figure 1, D–F). Upon tail-vein injection, 4T1-SCA1^+^ cells displayed significantly greater lung colonization ability compared with 4T1-SCA1^–^ cells (Figure 1, G–I). In addition, 4T1-SCA1^+^ cells showed significantly higher in vitro mammosphere-forming efficiency than 4T1-SCA1^–^ cells (Supplemental Figure 2A), while in vitro cell growth and cell motility were comparable (Supplemental Figure 2, B and C). These results suggest that 4T1-SCA1^+^ and 4T1-SCA1^–^ cells have similar tumorigenic potential, while 4T1-SCA1^+^ cells have higher mammosphere-forming and metastasis-initiating capacities.

### The SCA1^+^ tumor cell population is plastic in vitro and in vivo.

Growing evidence indicates that cancer cells possess plastic features modulated by both cell-intrinsic factors and microenvironmental cues (39, 40). To characterize the observed plasticity of 4T1-SCA1^+^ cells, we first investigated isolated 4T1-SCA1^+^ and 4T1-SCA1^–^ cells in vitro. The frequency of the SCA1^+^ population in MACS-enriched 4T1-SCA1^+^ cells (>93%) gradually decreased to around 50% after 4 days of culture, while the SCA1^–^ cells (>99% negatively enriched by MACS) regenerated a SCA1^+^ population (from <1% to >20%) (Supplemental Figure 2D). Consistently, after orthotopic injection of 4T1-SCA1^+^ cells, the abundance of the SCA1^+^ population from the derived tumors decreased from 85% after MACS enrichment at injection time to about 40% after 23 days of in vivo growth, while in tumors generated from 4T1-SCA1^–^ cells, it increased from less than 1% to 15% (Supplemental Figure 2E), similar to the frequency of SCA1^+^ population in tumors derived from parental 4T1 cells (Figure 1C). When tumor cells derived from PTs and lung metastases of parental 4T1–injected mice were cultured ex vivo, the abundance of the SCA1^+^ population significantly decreased, from 20% to 4.5% and from 60% to 19%, respectively (Supplemental Figure 2F). From these observations, we conclude that both SCA1^+^ and SCA1^–^ populations exhibit high plasticity, which can be modulated by the TME in vivo.

### Tu-Gr1^+^CD11b^+^ cells demonstrate higher potency in expanding the metastatic SCA1^+^ population.

**Figure 2:**
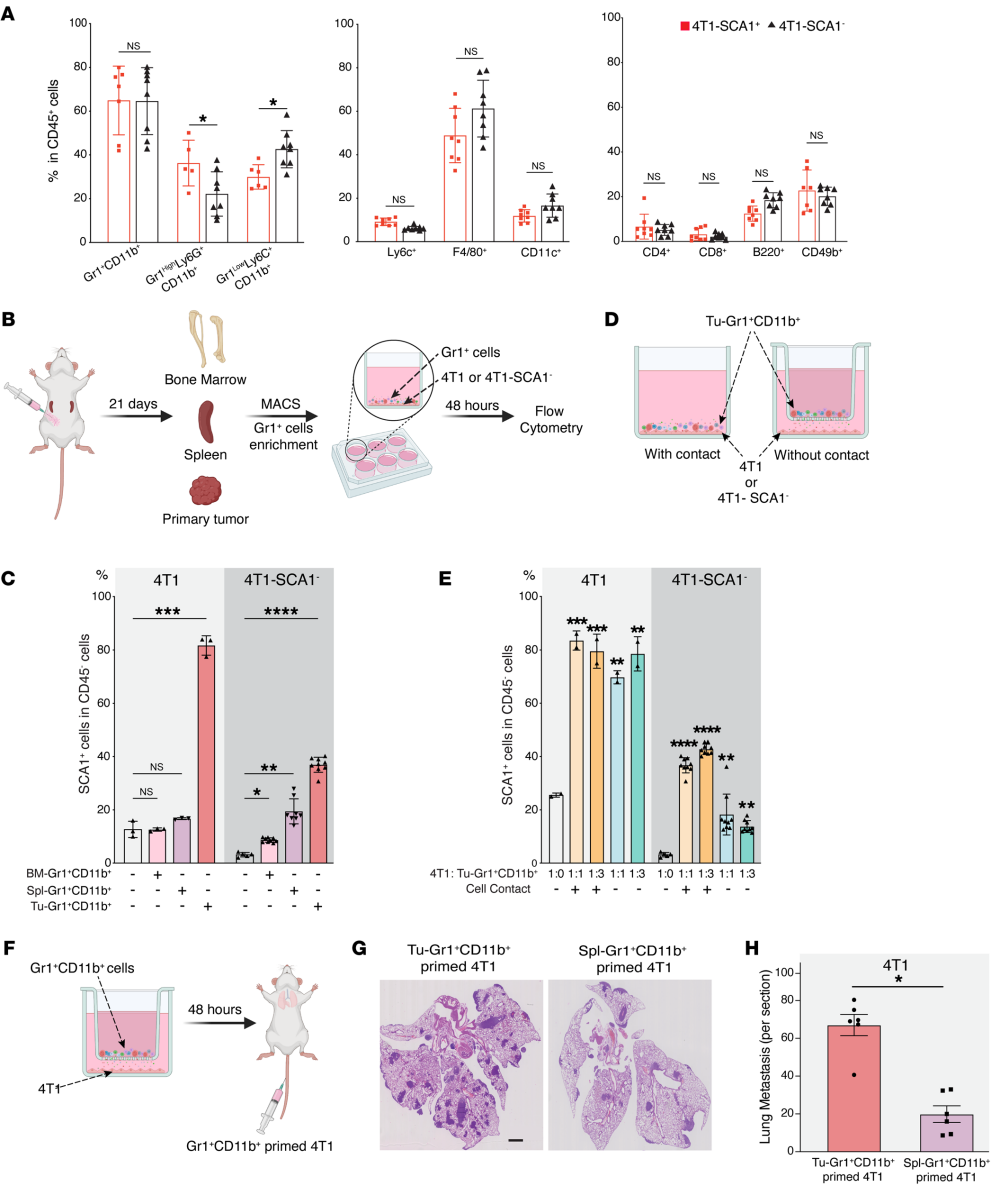
SCA1 expression is modulated by TME. (**A**) Frequency of different immune cell populations in PTs of mice orthotopically injected with 4T1-SCA1^+^ and 4T1-SCA1^–^ cells 21 days after injection. Populations are determined in CD45-positive, viable cells. *n* = 8 mice/group. (**B** and **C**) Schematic (**B**) showing experimental design for isolating Gr1^+^ cells from different sites of tumor-bearing mice. Twenty-one days after tumor implantation, Gr1^+^ cells were isolated from bone marrow (BM-Gr1^+^CD11b^+^), spleen (Spl- Gr1^+^CD11b^+^), or PT (Tu-Gr1^+^CD11b^+^) and cocultured for 48 hours with parental 4T1 or sorted 4T1-SCA1^–^ cells in vitro. SCA1 expression in tumor cells was examined by flow cytometry (**C**). Coculture conditions are indicated in bar graph. *n* = 3/group for 4T1; *n* = 5–9/group for 4T1-SCA1^–^. (**D** and **E**) Schematic of experimental coculture setup (**E**). MACS-enriched Gr1^+^ cells were cocultured with 4T1 or sorted 4T1-SCA1^–^ cells with or without Transwell inserts of 0.4 μm pore size. Cells were seeded in bottom well and Gr1^+^CD11b^+^ cells in upper part of insert. After 48 hours, tumor cells were examined for SCA1 expression by FACS (**E**). Coculture conditions are indicated in bar graph. Ratio of tumor cells and Tu-Gr1^+^CD11b^+^ varied from 1:1 to 1:3. *n* = 3/group for 4T1; *n* = 5–9/group for 4T1-SCA1^–^. (**F**–**H**) Schematic of experimental metastasis setup for evaluation of metastatic capacity of Gr1^+^CD11b^+^-educated 4T1 cells in vivo (**F**). 4T1 tumor cells were primed with Tu-Gr1^+^CD11b^+^ or Spl-Gr1^+^CD11b^+^ in vitro without cell-cell contact for 48 hours and injected into tail veins. Lung metastases were quantified 10 days after injection (**G**). Representative H&E staining images of lung sections are shown (**H**). Scale bar: 1 mm. *n* = 6 mice/group. Data are represented as means ± SEM and are representative of 3 independent experiments. *P* values were calculated using unpaired 2-tailed Student’s *t* test (**A** and **H**) or 1-way ANOVA with Dunnett’s multiple-comparison test (**C** and **E**). *P < 0.05; **P < 0.01; ***P < 0.001; ****P < 0.0001.

Immune cells in the TME are critical determinants of tumor cell behavior, including metastatic capacity (41). To collect evidence for a potential correlation between immune cells and the SCA1^+^ population, we characterized the inflammatory cells infiltrating the PTs following orthotopic injection of 4T1-SCA1^+^ and 4T1-SCA1^–^ cells. We observed a significant increase of the Gr1^hi^Ly6G^+^Ly6C^lo^CD11b^+^ population and a significant decrease of the Gr1^lo^Ly6G^–^Ly6C^hi^ CD11b^+^ population in tumors from 4T1-SCA1^+^– compared with 4T1-SCA1^–^–injected mice (Figure 2A). To examine the direct contribution of the Tu-Gr1^+^CD11b^+^ cells in promoting the enrichment of the SCA1^+^ population, we MACS isolated Gr1^+^ cells from PTs, bone marrow, and spleen of 4T1 tumor–bearing BALB/c mice and cocultured them in vitro with parental 4T1 or sorted 4T1-SCA1^–^ cells (Figure 2B). Gr1^+^ cells isolated from tumors (Tu-Gr1^+^CD11b^+^) induced a significant expansion of the SCA1^+^ population from both 4T1 and 4T1-SCA1^–^ cells within 48 hours (Figure 2C). In contrast, Gr1^+^ cells from the spleen (Spl-Gr1^+^CD11b^+^) or bone marrow (BM-Gr1^+^CD11b^+^) were less efficient and induced the SCA1^+^ population only from 4T1-SCA1^–^ cells at lower frequency (between 10% and 20%), comparable to unsorted 4T1 cells (Figure 2C). Interestingly, the Tu-Gr1^+^CD11b^+^–induced SCA1^+^ population appeared more stable in time compared with the isolated 4T1-SCA1^+^ cells (Supplemental Figure 2G). Moreover, Tu-Gr1^+^CD11b^+^ cells, rather than BM-Gr1^+^CD11b^+^ or Spl-Gr1^+^CD11b^+^ cells, were more effective in expanding the SCA1^+^ population in D2A1 cells, which have low intrinsic frequency of SCA1^+^ cells (4%–5%) (Supplemental Figure 3, A and B).

To determine whether the expansion of a SCA1^+^ population requires direct contact with Tu-Gr1^+^CD11b^+^ or is mediated by soluble factors, we compared the induction in standard wells (contact) versus Transwells (without contact) (Figure 2D). There was no significant difference in the expansion of the SCA1^+^ population between these 2 conditions in 4T1 cells. Additionally, increasing the ratio of 4T1 to Tu-Gr1^+^CD11b^+^ cells from 1:1 to 1:3 did not lead to further expansion of the SCA1^+^ population. Similar results were observed in 4T1-SCA1^–^ cells, which, however, appeared to rely more on cell-cell contact conditions (Figure 2E). Tu-Gr1^+^CD11b^+^ cells were also able to expand the SCA1^+^ population in D2A1 cells contact free in a dose-dependent manner (Supplemental Figure 3C). Importantly, when Tu-Gr1^+^CD11b^+^ cell–primed 4T1 cells were injected into the tail vein, they formed more lung metastases compared with Spl-Gr1^+^CD11b^+^ cell–primed 4T1 cells (Figure 2, F–H).

Tu-Gr1^+^CD11b^+^ is a mixed cell population, including Gr1^hi^Ly6G^+^Ly6C^lo^CD11b^+^ tumor-associated neutrophils (TANs) and Gr1^lo^Ly6G^–^Ly6C^+^CD11b^+^ monocytic cells. Both of these cell populations originate from Gr1^+^CD11b^+^ immature myeloid progenitors mobilized from the bone marrow to the TME and promote tumor progression and metastasis (42, 43). Their surface markers broadly overlap, impairing phenotypical distinction (43). In tumor-bearing mice, we observed homogenous Gr1^+^CD11b^+^ cells in the circulation, which is consistent with a previous report (44), while in the TME, they differentiated into Gr1^hi^ and Gr1^lo^ subpopulations in both 4T1- and D2A1-injected mice (Supplemental Figure 3D). To elucidate differences in their capacity to induce the SCA1^+^ population, we isolated Gr1^hi^CD11b^+^ and Gr1^lo^CD11b^+^ cells from PTs by FACS sorting and cocultured them with 4T1 or 4T1-SCA1^–^ cells (Supplemental Figure 3E). Both Gr1^hi^ and Gr1^lo^ subpopulations demonstrated the ability to expand the SCA1^+^ population, with Gr1^hi^CD11b^+^ cells being more potent (Supplemental Figure 3F). Together, these results imply that Tu-Gr1^+^CD11b^+^ cells induce the emergence of a highly metastatic SCA1^+^ population through secreted factors.

### Tu-Gr1^+^CD11b^+^–induced and tumor-inherent SCA1^+^ populations display distinct gene expression profiles.

**Figure 3:**
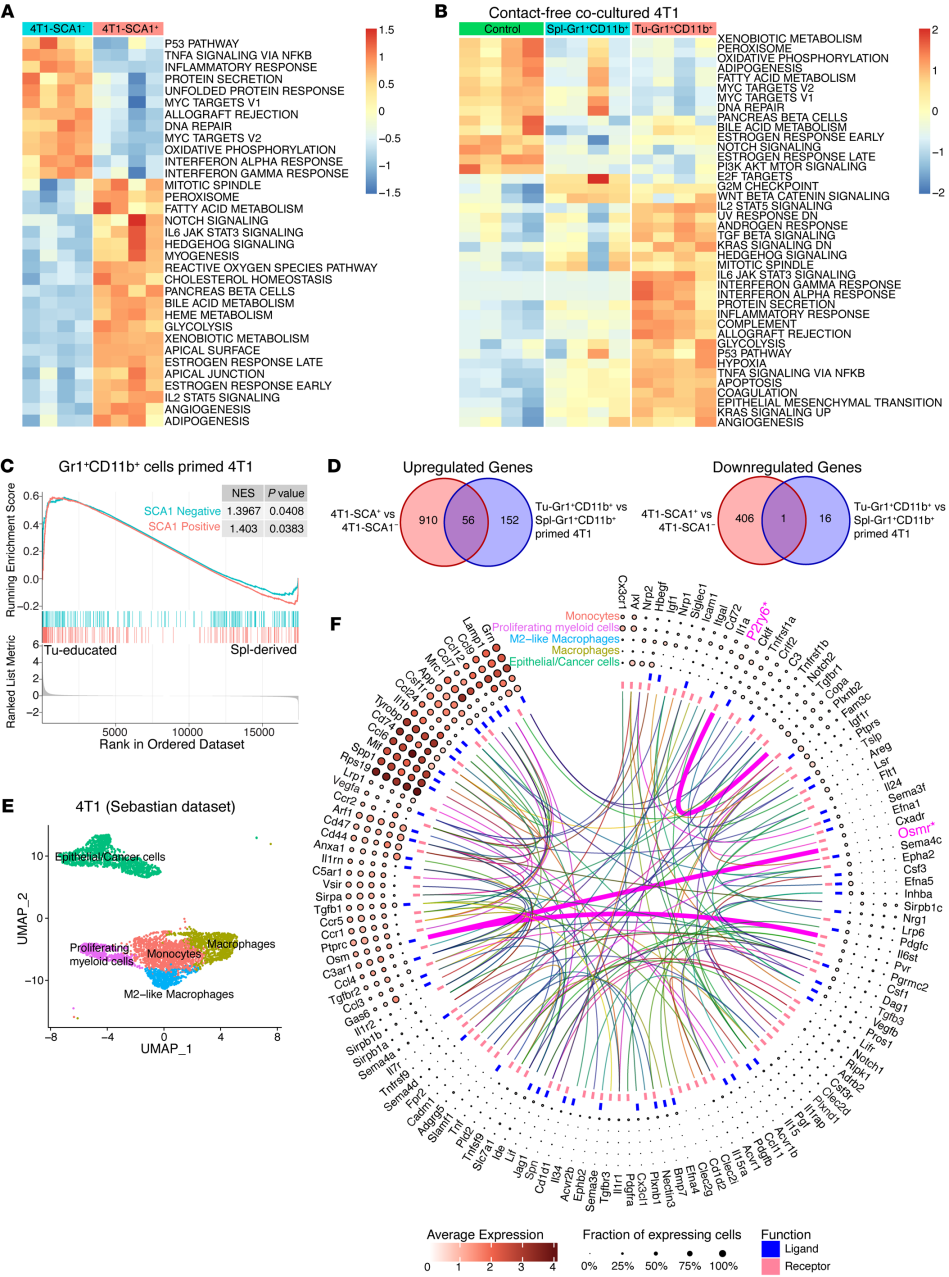
Transcriptomic analysis of SCA1^+^ tumor cells. (**A**) Heatmap showing the signature score of the hallmark pathways analysis in 4T1-SCA1^+^ and 4T1-SCA1^–^ populations sorted from parental 4T1 cells. The colors code the expression levels relative to average levels, as indicated at the bottom. (**B**) Heatmap showing the signature score of the hallmarks pathway analysis in parental 4T1, Spl-Gr1^+^CD11b^+^–primed 4T1, and Tu-Gr1^+^CD11b^+^–primed 4T1 cells. The colors code the expression levels relative to average levels, as indicated at the bottom. (**C**) GSEA comparing the Tu-Gr1^+^CD11b^+^– and Spl-Gr1^+^CD11b^+^–primed 4T1 cells. GSEA shows positive correlations of both SCA1-positive and SCA1-negative signatures. NES, normalized enrichment score. (**D**) Venn diagrams showing that 56 upregulated genes and 1 downregulated gene are shared between inherent and Tu-Gr1^+^CD11b^+^–induced SCA1^+^ population in 4T1 tumor cells. (**E**) UMAP plot showing clusters of cancer cells and myeloid cell populations in orthotopically grown 4T1-derived PTs extracted from the Sebastian data set (see Methods for details). (**F**) Circos diagram showing the predicted potential interactions between cancer cells and different myeloid cell populations determined by CellPhoneDB (see Supplemental Methods for details) based on the Sebastian data set. Only *Osmr* and *P2ry6* are shared with the common 56 gene list shown in panel **D**.

To unravel the molecular basis for the metastatic capacity of the inherent 4T1-SCA1^+^ population and the Tu-Gr1^+^CD11b^+^–induced SCA1^+^ population, we first performed transcriptomic profiling of 4T1-SCA1^+^ and 4T1-SCA1^–^ cells isolated from the parental 4T1 line. Pathway enrichment analysis showed that 4T1-SCA1^+^ and 4T1-SCA1^–^ cells expressed genes associated with distinct signaling pathways (Figure 3A). The top 200 significantly upregulated and downregulated genes were extracted as SCA1-positive and SCA1-negative signatures, respectively (Supplemental Table 1). Next, we performed transcriptomic profiling of Tu-Gr1^+^CD11b^+^–primed 4T1, Spl-Gr1^+^CD11b^+^-primed 4T1, and parental 4T1 cells. Pathway enrichment analysis revealed that Tu-Gr1^+^CD11b^+^ and Spl-Gr1^+^CD11b^+^ priming induced distinct transcriptomic alterations in 4T1 cells (Figure 3B). Comparative analysis revealed that Tu-Gr1^+^CD11b^+^–primed 4T1 cells expressed both SCA1-positive and SCA1-negative signatures (Figure 3C), suggesting that Tu-Gr1^+^CD11b^+^ may twist tumor plasticity by converting the 4T1-SCA1^–^ cells into 4T1-SCA1^+^ cells, rather than expanding the preexisting 4T1-SCA1^+^ population.

To test for possible common molecular mechanisms underlying the induction and metastatic capacity of the different SCA1^+^ populations, we compared the significantly differentially expressed genes (DEG) between 4T1-SCA1^+^ versus 4T1-SCA1^–^ and Tu-Gr1^+^CD11b^+^–primed 4T1 versus Spl-Gr1^+^CD11b^+^–primed 4T1 cells. Strikingly, among a total of 1118 upregulated and 423 downregulated DEGs in the 2 conditions, only 56 upregulated and 1 downregulated genes were shared (Figure 3D and Supplemental Table 1). This was consistent with the notion that the SCA1^+^ population in Tu-Gr1^+^CD11b^+^–primed 4T1 cells was different from the inherent 4T1-SCA1^+^ cells. Nonetheless, the fact that the 4T1-SCA1^+^ cells and Tu-Gr1^+^CD11b^+^–primed 4T1 cells possessed similar in vivo metastatic capacities suggested that, among these common pathways, some were relevant for 4T1 metastasis formation. To address this question, we analyzed the scRNA-Seq data set from 4T1 PTs of Sebastian et al. (45) encompassing several cell types, including cancer cells, epithelial cells, fibroblasts, and distinct subpopulations of myeloid cells. To determine relevant ligand-receptor interactions between epithelial/cancer cells and myeloid/monocytic cells, we performed cell-cell interaction analysis from the scRNA-Seq data with CellPhoneDB (46) (Figure 3, E and F). The analysis identified 160 ligand-receptor interaction pairs (Supplemental Table 2). Among those pairs, OSM receptor (*OSMR*) and pyrimidinergic receptor P2Y6 (*P2RY6*) were the only ones present among the 56 common genes shown in Figure 3D. However, P2RY6 interacts with coatomer complex subunit α (COPA), a membrane protein involved in membrane traffic between endoplasmic reticulum and Golgi (47) and thus unlikely to mediate cell-cell contact-independent induction of the SCA1^+^ population. As the IL-6/JAK/STAT3 signaling pathway was upregulated both in the 4T1-SCA1^+^ population and Tu-Gr1^+^CD11b^+^–primed 4T1 cells (Figure 3, A and B), we examined the expression of *Osm*, *Osmr*, *Il6st*, *Il6*, and *Il6* receptor (*Il6ra*) in the Sebastian data set (Supplemental Figure 4A). The expression of *Il6* and *Osm* was restricted to myeloid cells, with *Osm* expression being more prominent, consistent with a previous report (34). *Osmr* was predominantly expressed in tumor cells, while *Il6st* and *Il6ra* were homogenously expressed in all cell types.

In contrast, *Osm* and *Il6* expression were very low in all 4T1-SCA1^+^ cells and Tu-Gr1^+^CD11b^+^–primed 4T1 cell–related samples (normalized count number less than 7 on average) (Supplemental Figure 4, B and C). *Osmr* and *Il6ra*, however, were highly expressed in 4T1-SCA1^+^ cells compared with 4T1-SCA1^–^ cells, while the expression of *Il6st* was abundant in both populations (Supplemental Figure 4B). Meanwhile, *Osmr* was significantly upregulated in Tu-Gr1^+^CD11b^+^–primed 4T1 compared with Spl-Gr1^+^CD11b^+^-primed 4T1 (Supplemental Figure 4C). Taken together, these results suggest that OSM/OMSR and IL-6/IL-6R signaling pathways may be involved in expansion of the SCA1^+^ population mediated by Tu-Gr1^+^CD11b^+^ cells.

### Tu-Gr1^+^CD11b^+^ cells promote SCA1^–^ to SCA1^+^ population conversion.

**Figure 4:**
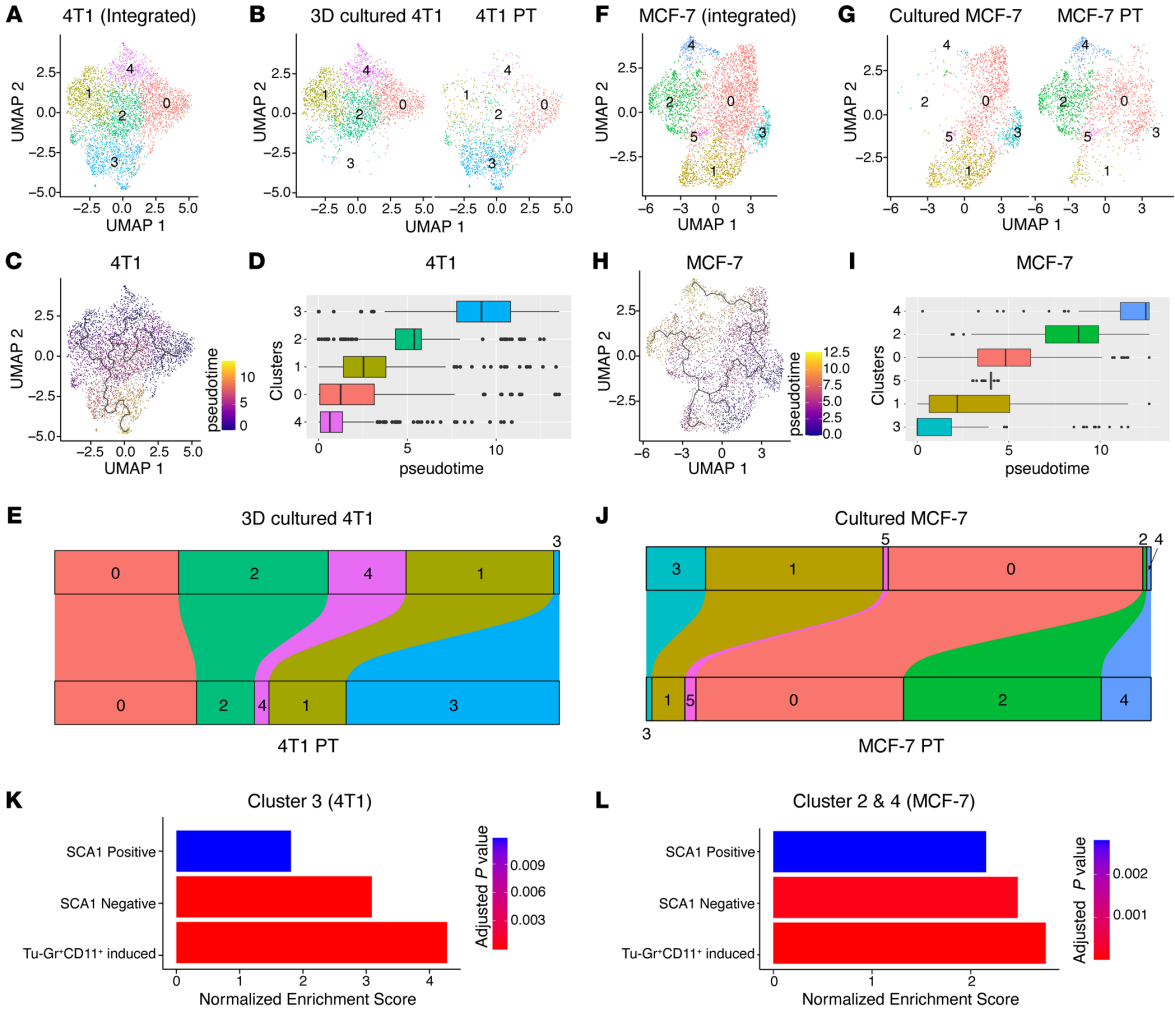
Transformation dynamics of tumor cell populations induced by the TME. (**A**) UMAP plots showing 4T1 clusters based on integrated scRNA-Seq data from 4T1 cells in 3D culture or in PT. (**B**) Distribution of specific clusters in 4T1 cells in 3D culture or PT. (**C** and **D**) UMAP plot (**C**) and box plot (**D**) showing the clusters in pseudo-time course during the transformation of 4T1 cells from ex vivo culture to in vivo. (**E**) Sankey diagram showing the dynamic of each cluster during the transformation of 4T1 cells from ex vivo culture to in vivo. Cluster 3 was largely expanded in vivo. (**F**) UMAP plots showing MCF-7 clusters based on integrated scRNA-Seq data from MCF-7 cells in culture or in PT. (**G**) Distribution of the specific clusters in cultured MCF-7 cells or MCF-7 PTs. (**H**–**I**) UMAP plot (**H**) and box plot (**I**) showing the clusters in pseudo-time course during the transformation of MCF-7 cells from ex vivo culture to in vivo. (**J**) Sankey diagram showing the dynamic of each cluster during the transformation of MCF-7 cells from ex vivo culture to in vivo. Clusters 2 and 4 were largely expanded in vivo. (**K** and **L**) GSEA analysis of SCA1-positive signature, SCA1-negative signature, and Tu-Gr1^+^CD11b^+^–induced signature of cells in cluster 3 in 4T1 data (**K**) and in cluster 2 and cluster 4 in MCF-7 data (**L**). Analyses are based on publicly available data (4T1: GEO GSM4812003 and GSM3502134; MCF-7: GEO GSM4681765 and GSM5904917).

The above results strongly implied that Tu-Gr1^+^CD11b^+^ cells convert SCA1^–^ cells into the SCA1^+^ population. To further test this hypothesis, we compared the cell population dynamics in cultured cells and the orthotopic PTs by analyzing publicly available scRNA-Seq data sets. By integrating scRNA-Seq data from 3D cultured 4T1 cells (NCBI’s Gene Expression Omnibus [GEO] GSM4812003) (48) and tumor cells isolated from orthotopically injected 4T1 PTs (GEO GSM3502134) (49) (Figure 4, A and B), we observed 5 clusters: clusters 0, 1, 2, and 4 were predominant in cultured tumor cells, while cluster 3 was predominant in PT. Single-cell trajectories analysis confirmed that cluster 3 was at the end of the transformation process (Figure 4, C and D). The population dynamics also showed that the fraction of cells in clusters 1, 2, and 4 decreased during the transformation and the one in cluster 0 only minimally increased, while the fraction in cluster 3 massively increased (Figure 4E). Importantly, very few cultured 4T1 cells expressed *Sca1*, while it was abundantly expressed in most cells in the PT (Supplemental Figure 5A). Consistently, the fraction of cells expressing *Osmr* was higher in the PT compared with cultured cells (Supplemental Figure 5A). Similar observations were obtained when analyzing scRNA-Seq data from the ER^+^ human breast cancer model MCF-7. After integrating data from cultured MCF-7 cells (GEO GSM4681765) and MCF-7 cells that were isolated from tumors generated by MCF-7 cells injected in the mammary gland intraductally (GEO GSM5904917) (50), 6 clusters were identified (Figure 4F), with clusters 1 and 3 predominant in cultured MCF-7 cells, while clusters 2 and 4 were prevalent in PTs (Figure 4G). Clusters 2 and 4 expanded during in vitro–to–in vivo tumor cell transformation and represented nearly 50% of the in vivo PT cells (Figure 4, H–J). Although there is no human homolog of the *Sca1* gene for comparison, *OSMR*-expressing cells were increased upon tumor implantation, especially in clusters 2 and 1 (Supplemental Figure 5B).

To investigate the signals involved in this transformation, we performed Gene Set Enrichment Analysis (GSEA) for cluster 3 in 4T1 cells and clusters 2 and 4 in MCF-7 cells, respectively (Figure 4, K and L, and Supplemental Figure 5, C and D). By comparing the Hallmark gene signatures, the IL-6/JAK/STAT3 signature was significantly upregulated in both cell populations (Supplemental Figure 5, C and D). Interestingly, SCA1-positive and SCA1-negative signatures were both upregulated (Figure 4, K and L), coherently with our ex vivo induction experiment (Figure 3C). To validate the involvement of Tu-Gr1^+^CD11b^+^ during the cell population transformation, we extracted the top 50 upregulated genes (Supplemental Table 3) identified by comparing the Tu-Gr1^+^CD11b^+^– with the Sp-Gr1^+^CD11b^+^–stimulated 4T1 cells as Tu-Gr1^+^CD11b^+^–induced signature. The clusters predominant in the PT in both 4T1 (cluster 3) and MCF-7 (clusters 2 and 4) models showed an upregulation of the Tu-Gr1^+^CD11b^+^–induced signature (Figure 4, K and L). These data, together with the ex vivo coculture experiments (Figure 2, B–G, and Figure 3C), demonstrate that Tu-Gr1^+^CD11b^+^ cells convert the SCA1^–^ population into SCA1^+^ population, possibly via the OSM/IL-6 signaling pathway.

### OSM/IL-6/JAK pathway mediates Tu-Gr1^+^CD11b^+^–induced SCA1^+^ population enrichment.

**Figure 5:**
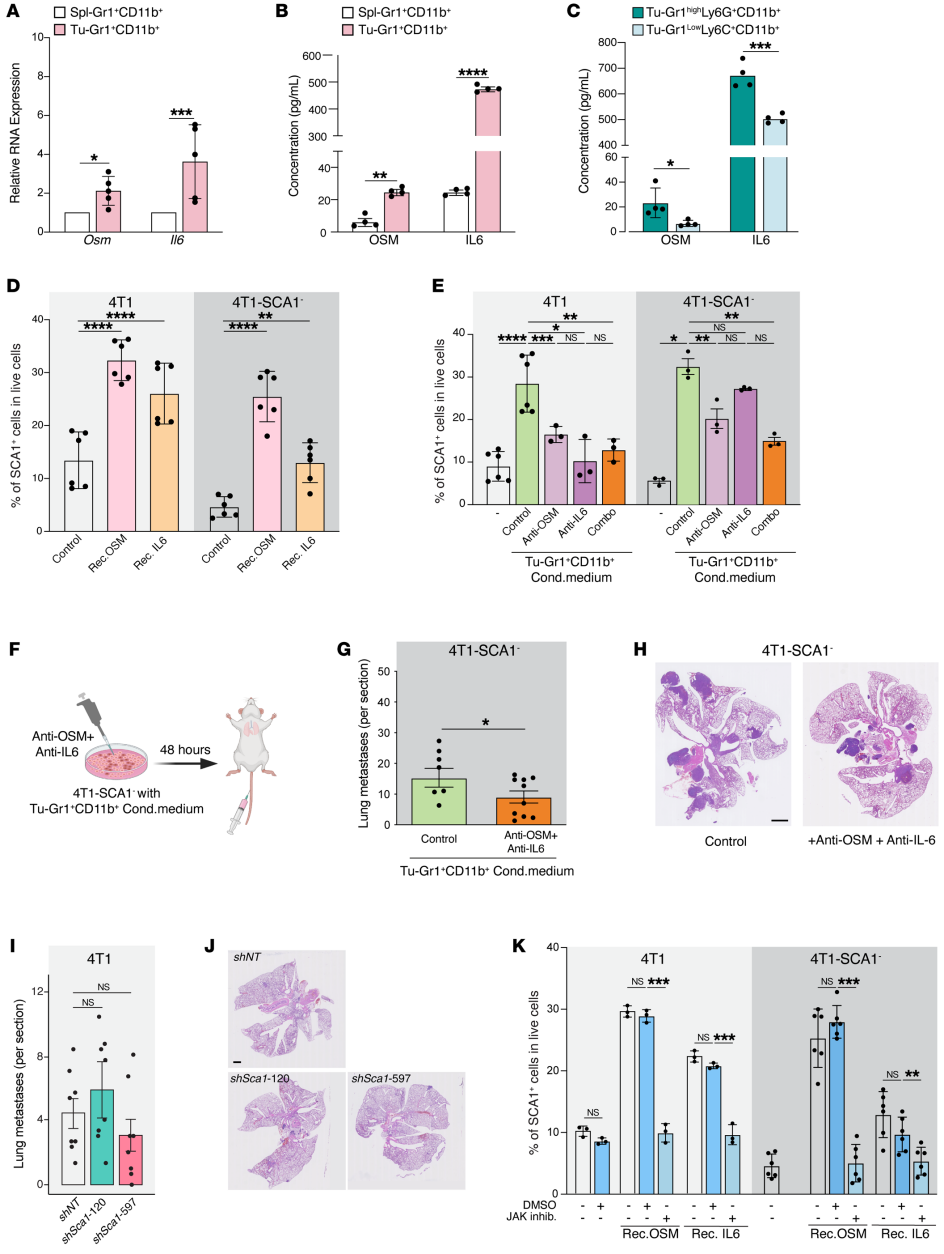
SCA1^+^ population is modulated by OSM/IL-6/JAK pathway. (**A**) Relative *Osm* and *Il6* mRNA expression in Tu-Gr1^+^CD11b^+^ and Spl-Gr1^+^CD11b^+^ cells. *n* = 4–5/group. (**B** and **C**) OSM and IL-6 protein quantification in supernatant of (**B**) Tu-Gr1^+^CD11b^+^ and Spl-Gr1^+^CD11b^+^ (**C**) and Tu-Gr1^hi^Ly6G^+^CD11b^+^ and Gr1^lo^Ly6G^–^CD11b^+^ cells. *n* = 4/group. (**D**) Fraction of SCA1^+^ cells of parental 4T1 or sorted 4T1-SCA1^–^ cells upon exposure to OSM or IL-6 (10 ng/ml 48 hours). *n* = 6/group. (**E**) Effect of inhibition of OSM and IL-6 in Tu-Gr1^+^CD11b^+^ conditioned medium on SCA1 expression in parental 4T1 or sorted 4T1-SCA1^–^ cells after 48 hours treatment. *n* = 3–6/group. (**F**) Experimental design for examining lung metastatic capacity of IL-6/OSM. 4T1-SCA1^–^ cells were primed by Tu-Gr1^+^CD11b^+^ conditioned medium with dual depletion of OSM/IL-6 depleted or control, in vitro for 48 hours and then injected into tail vein. Lungs were examined for metastasis 10 days later. (**G** and **H**) Quantification of lung metastases (**G**) and representative H&E staining images of lung sections (**H**). *n* = 8–10/group. Scale bar: 1 mm. (**I** and **J**) Quantification of lung metastases in mice injected with 4T1 control and 4T1 silenced for *Sca1* (*shSca1-120* and *shSca1-597*) (**I**). *n* = 8/group. Representative H&E stained lung sections (**J**). Scale bar: 1 mm. (**K**) SCA1^+^ population stimulation in cultured parental 4T1 or sorted 4T1-SCA1^–^ cells by recombinant OSM or IL-6 (10 ng/ml, 48 hours) in vitro in presence of ruxolitinib (5 μM) or DMSO control. *n* = 3–6/group. Data are represented as means ± SEM from 3 independent experiments. *P* values were calculated using unpaired 2-tailed Student’s *t* test (**A**–**C** and **G**); unpaired 2-tailed Student’s *t* test with Holm’s correction (**I**); 1-way ANOVA with Dunnett’s multiple-comparison test (**D** and **K**); or 1-way ANOVA with Tukey’s multiple-comparison test (**E**). *P < 0.05; **P < 0.01; ***P < 0.001; ****P < 0.0001.

To experimentally interrogate the role of OSM/IL-6 in modulating the SCA1^+^ population, we first measured *Osm* and *Il6* mRNA expression by quantitative PCR (qPCR) in Spl-Gr1^+^CD11b^+^ and Tu-Gr1^+^CD11b^+^ cells and protein levels by ELISA in conditioned supernatants. Indeed, we found that the expression of *Osm* and *Il6* in both mRNA and protein levels was significantly elevated in Tu-Gr1^+^CD11b^+^ cells (Figure 5, A and B). In addition, higher OSM and IL-6 protein secretion were observed in Tu-Gr1^hi^Ly6G^+^CD11b^+^ cells compared with Tu-Gr1^lo^Ly6C^+^CD11b^+^ cells (Figure 5C), correlating with their ability to induce 4T1-SCA1^+^ cells (Supplemental Figure 3E).

To functionally validate the role of OSM/IL-6 in promoting the SCA1^+^ population, we first treated 4T1, 4T1-SCA1^–^, or D2A1 cells for 2 days with recombinant OSM and IL-6 proteins in vitro. Both treatments significantly increased the frequency of the SCA1^+^ population (Figure 5D and Supplemental Figure 6A). To validate the functional role of OSM and IL-6 produced by Tu-Gr1^+^CD11b^+^ cells, we set up an inhibition experiment. First, we confirmed that the conditioned medium produced by Tu-Gr1^+^CD11b^+^ cocultured with 4T1 cells retained the ability to enhance the SCA1^+^ population in 4T1 or 4T1-SCA1^–^ cells (Supplemental Figure 6, B and C). Next, we depleted OSM and IL-6 in the Tu-Gr1^+^CD11b^+^ conditioned medium using neutralizing antibodies against OSM or IL-6 and cultured with 4T1 cells, resulting in a significant reduction of the emergence of the SCA1^+^ population from 4T1 or 4T1-SCA1^–^ cells (Figure 5E). The combination of anti-OSM and anti–IL-6 antibodies did not have additive effects, suggesting that OSM and IL-6 contribute to promote the SCA1^+^ population through the same pathway. Importantly, tail-vein injection of 4T1-SCA1^–^ cells treated ex vivo with OSM/IL-6–depleted Tu-Gr1^+^CD11b^+^ conditioned medium blunted its prometastatic effect in vivo, thus demonstrating the role of Tu-Gr1^+^CD11b^+^–derived OSM/IL-6 in promoting metastasis (Figure 5, F–H).

Next, we asked whether SCA1 itself functionally contributes to the OSM/IL-6–induced metastatic population in 4T1 cells. To this end, we stably silenced SCA1 in 4T1 cells by lentiviral-mediated shRNA delivery. The *Sca1* silencing efficiency was validated via OSM treatment in vitro (Supplemental Figure 6D). *Sca1* silencing with 2 independent shRNAs did not affect PT growth upon orthotopic cell injection nor did it reduce lung metastasis formation, indicating that SCA1 itself is not the mediator of metastasis (Figure 5, I and J, and Supplemental Figure 6, E and F).

OSMR and IL-6R signal by activating the intracellular JAK (51). To explore the involvement of the JAK pathway in the emergence of the SCA1^+^ population, we treated tumor cells with a JAK inhibitor (ruxolitinib) during exposure to recombinant OSM and IL-6. Ruxolitinib treatment prevented the emergence of the SCA1^+^ population in response to recombinant OSM and IL-6 in 4T1, 4T1-SCA1^–^, and D2A1 cells (Figure 5K and Supplemental Figure 6G). From these data, we conclude that the OSM/IL-6/JAK pathway has a crucial role in mediating the enrichment of the highly metastatic SCA1^+^ population induced by Tu-Gr1^+^CD11b^+^.

### Tu-Gr1^+^CD11b^+^–induced SCA1^+^ population and 4T1-inherent SCA1^+^ population have distinct CSC and EMT gene expression profiles.

OSM/IL-6/JAK signaling has been reported to support tumor progression by promoting a CSC phenotype and epithelial-mesenchymal plasticity (26, 37, 52). To characterize CSC and EMT features in 4T1-SCA1^+^ cells and a Tu-Gr1^+^CD11b^+^–induced SCA1^+^ population, we interrogated our RNA-Seq data sets for the expression of 9 stem cell and 8 EMT marker genes (Supplemental Figure 7). The expressions of the stem cell markers *Oct4* (*Pou5f1*), *Sox2*, and *Nanog* were undetectable or very low in all (or some) samples. 4T1-SCA1^+^ cells showed higher expression of *Aldh1a1*, *Aldh3a1*, and *Podxl*, but lower expression of *Klf4* and *Sox9* compared with 4T1-SCA1^–^ cells. There was no difference in the expression of *Abcg2* and *Has2*. Tu-Gr1^+^CD11b^+^–primed 4T1 cells had higher expression of *Klf4* and *Has2*, lower expression of *Aldh1a1*, *Aldh3a1*, and *Sox9*, and similar expression of *Podxl* when compared with Spl-Gr1^+^CD11b^+^–primed 4T1 cells. Among them, only *Has2* expression was specifically elevated in Tu-Gr1^+^CD11b^+^–primed 4T1 cells compared with control 4T1 and Spl-Gr1^+^CD11b^+^–primed 4T1 cells (Supplemental Figure 7A). On the other hand, 4T1-SCA1^+^ cells had lower expression of *Cdh1* and higher expression of *Snail1*, *Twist1*, *Vim*, and *Foxc1*, which support an EMT status, although *Zeb1* expression was reduced (Supplemental Figure 7B). Globally, the expression of most of the EMT genes was similar between Tu-Gr1^+^CD11b^+^– and Spl-Gr1^+^CD11b^+^–primed 4T1 cells, except for *Snail2* and *Vim*, whose expressions were suppressed in Tu-Gr1^+^CD11b^+^–primed 4T1 (Supplemental Figure 7B). Collectively, these results indicate that the Tu-Gr1^+^CD11b^+^–induced SCA1^+^ population and inherent SCA1^+^ population display different CSC and EMT transcriptional profiles, reinforcing the notion that Tu-Gr1^+^CD11b^+^–induced SCA1^+^ populations are not just enriched tumor-inherent SCA1^+^ populations.

### Chemotherapy enriches a SCA1^+^ population with CSC features.

**Figure 6:**
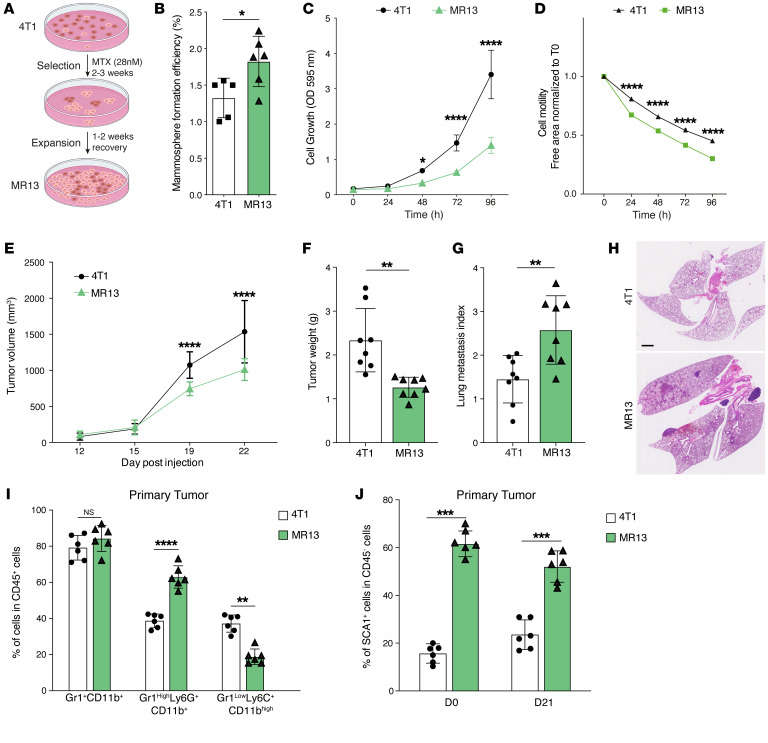
Long-term chemotherapy treatment of 4T1 cells induces a stable SCA1^+^ population (MR13) with higher metastatic capacity and CSC features. (**A**) Schematic of the experimental design to obtain chemotherapy-resistant MR13 cells from 4T1. (**B**) Quantification of the mammosphere-forming efficiency of 4T1 and MR13 tumor cells. *n* = 5–6/group. (**C**) Cell proliferation curves of 4T1 and MR13 tumor cells in vitro determined by the CV assay. Results are represented as mean of OD. *n* = 8/group. (**D**) Cell motility of 4T1 and MR13 tumor cells determined by the scratch wound healing assay. *n* = 5–6/group. Results are represented as cell-free area relative to the initial wound area from 3 independent experiments. (**E**) Growth curves of PTs in BALB/c mice orthotopically injected with 4T1 and MR13 tumor cells. *n* = 10–11/group. (**F**) Tumor weight of 4T1 and MR13 tumors recovered from BALB/c mice at day 22 after injection. *n* = 8/group. (**G** and **H**) Lung metastasis index 23 days after injection. The number of metastatic nodules is determined by H&E staining and normalized based on the PT weight (**G**). Representative H&E staining images of lung sections are shown (**H**). Scale bar: 1 mm. *n* = 8/group. (**I**) Frequency of different CD11b^+^ myeloid cells subpopulations in PTs from MR13- and 4T1-injected mice determined by flow cytometry 21 days after injection. *n* = 6/group. Subpopulations are determined in CD45-positive, viable cell population. (**J**) Percentage of SCA1^+^ tumor cells at time of injections (D0) of 4T1 and MR13 cells and in PTs recovered at day 21 (D21). SCA1 expression is determined in CD45-negative, viable cell population. *n* = 6/group. Data are represented as means ± SEM and are representative of 3 independent experiments. *P* values were calculated using unpaired, 2-tailed Student’s *t* test (**B**, **F**, **G**, **I** and **J**) or 2-way ANOVA with Tukey’s multiple comparison test for (**C**–**E**). *P < 0.05; **P < 0.01; ***P < 0.001; ****P < 0.0001.

CSC and cancer cell plasticity contribute to drug resistance in various tumor types (53–57). The above results, including the significantly elevated expression of *Aldh3a1* (a marker of drug resistance) in the 4T1-SCA1^+^ population, prompted us to investigate the resistance to chemotherapy of this population. To this end, we treated 4T1 cells for 48 hours in vitro with methotrexate (MTX) or doxorubicin (Dox), 2 widely used chemotherapy drugs. The 48-hour treatment with either drug increased the frequency of the SCA1^+^ population in 4T1 cells (Supplemental Figure 8A). To mimic the clinically relevant situation of cancer cells escaping chemotherapy treatment, we exposed 4T1 cells to a MTX concentration slightly higher than the IC_50_ of the drug (28 nM) for up to 3 weeks and recovered the surviving cells by switching to normal culture medium (Figure 6A). The selected MR13 cell line was highly enriched in SCA1^+^ cells (>60%) (Supplemental Figure 8B). Compared with parental 4T1 cells, MR13 cells exhibited a higher mammosphere-forming efficiency (Figure 6B), lower proliferative capacity (Figure 6C), and increased cell mobility (Figure 6D) in vitro, consistent with CSC-like properties. When tested in a 48-hour cytotoxicity assay, MR13 cells were more resistant to MTX compared with parental 4T1 (Supplemental Figure 8C).

### MR13-derived tumors are highly metastatic and enriched in Gr1^hi^Ly6G^+^CD11b^+^ cells.

To characterize the in vivo behavior of MR13 cells, we implanted them orthotopically into BALB/c mice. MR13 cells formed smaller PTs that were more metastatic to the lung (Figure 6, E–H) and enriched in Gr1^hi^Ly6G^+^CD11b^+^ cells compared with 4T1-derived tumors (Figure 6I), similarly to 4T1-SCA1^+^–derived tumors (Figure 2A). Strikingly, we observed metastases in the heart (Supplemental Figure 8D), which we never observed with parental 4T1 cells. MR13 cells retained a large fraction of the SCA1^+^ population in vitro, even when cultured in the absence of MTX (Supplemental Figure 8B) and upon in vivo expansion (Figure 6J). Such stability of the SCA1^+^ population contrasted with SCA1^+^ cells enriched by MACS in vitro or induced by the TME in vivo, both of which reverted to a SCA1^–^ phenotype shortly after in vitro culture (Supplemental Figure 2, D–F). This observation suggests that the MR13 line was capable of self-sustaining its own SCA1^+^ population. Taken together, these data show that chemotherapy-selected MR13 cells are stably enriched for the SCA1^+^ population both in vitro and in vivo and are highly metastatic in vivo.

### IL-6/IL-6R/JAK autocrine signaling maintains SCA1 positivity and metastatic capacity in MR13 cells.

**Figure 7:**
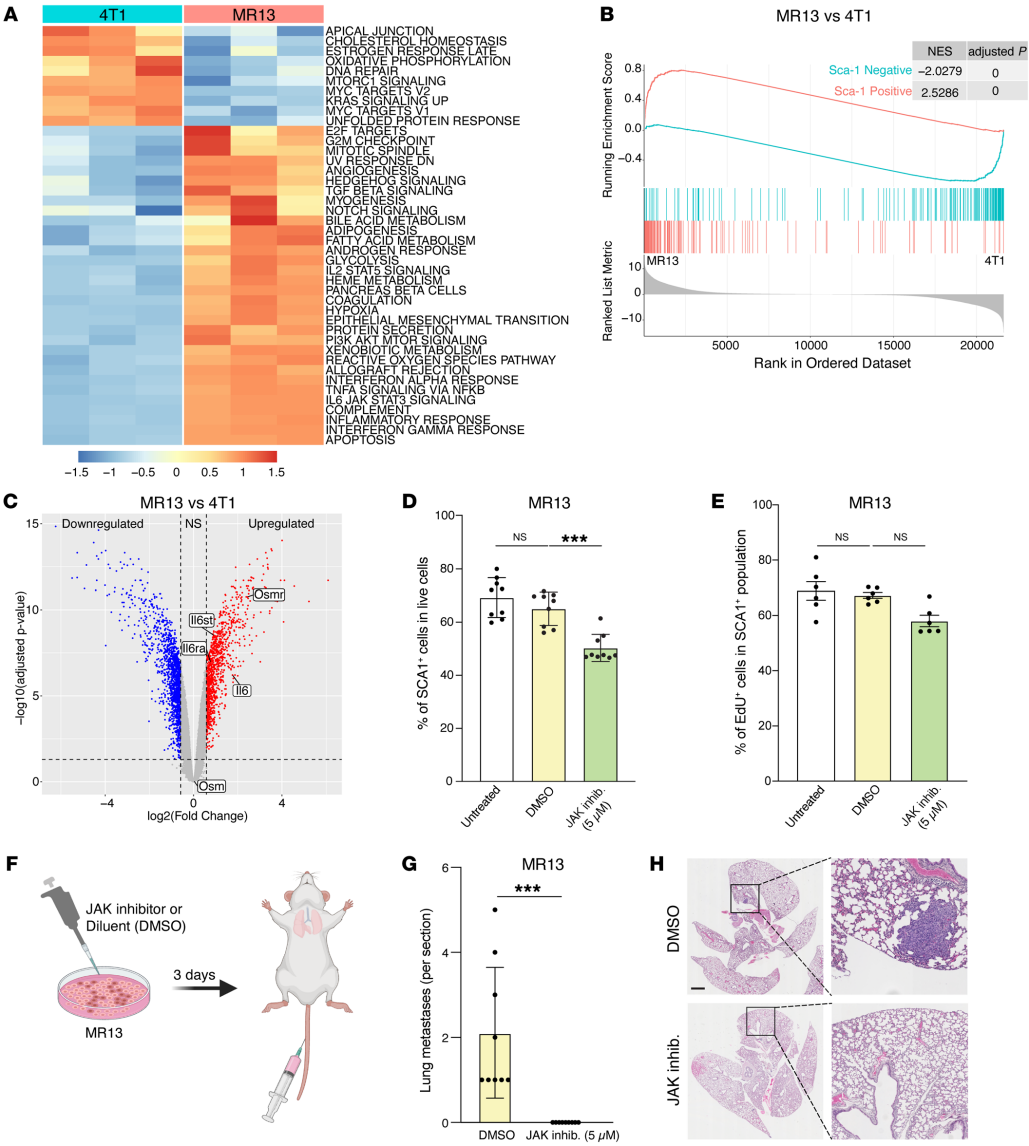
IL-6/JAK pathway promotes SCA1^+^ persistence and metastatic capacity in chemotherapy-resistant MR13 tumor cells. (**A** and **B**) Gene expression analysis of parental 4T1 and chemotherapy-resistant MR13 cells. Heatmap represents the signature score of the hallmark pathways analysis. Results from 3 biological replicates are shown (**A**). GSEA results show that MR13 cells are positively enriched for the SCA1-positive signature and negatively enriched for the SCA1-negative signature (**B**). (**C**) Volcano plot showing the differential expression of *Osm*, *Osmr*, *Il6st*, *Il6*, and *Il6ra* mRNA in MR13 versus 4T1 tumor cells. (**D**) Fraction of SCA1^+^ population in MR13 tumor cells treated for 72 hours with the JAK inhibitor ruxolitinib (5 μM) relative to vehicle control (DMSO) treatment. *n* = 9/group. (**E**) Percentage of EdU-positive in SCA1^+^ population of MR13 cells after treatment with JAK inhibitor ruxolitinib or DMSO control for 72 hours. *n* = 6/group. (**F**) Schematic of the experimental design for testing the effect of ruxolitinib on MR13 lung metastatic capacity shown in **G**–**H**. MR13 tumor cells were treated with ruxolitinib (5 μM) or DMSO in vitro for 72 hours and then injected into the mouse tail vein. Lungs were examined for metastasis 10 days after tumor cell injection. (**G** and **H**) Quantification of lung metastases in mice injected i.v. with MR13 treated in vitro with ruxolitinib (5 μM) or DMSO (**G**). Representative images of H&E staining are shown (**H**). *n* = 8/group. Scale bar: 1 mm. Data are represented as means ± SEM and are representative of 3 independent experiments for **D**, **E**, and **G**. *P* values were calculated using 1-way ANOVA with Dunnett’s multiple-comparison test (**D** and **E**) or unpaired 2-tailed Student’s *t* test (**G**). ***P < 0.001.

To better understand the chemotherapy-induced alterations in MR13 cells, we performed transcriptomic analyses comparing MR13 and parental 4T1 cells. Pathway enrichment analysis showed an upregulation of the IL-6/JAK/STAT3 signature in MR13 cells (Figure 7A). Importantly, MR13 gene expression significantly positively correlated with the SCA1-positive signature and negatively correlated with the SCA1-negative signature (Figure 7B). This suggested that chemotherapy enriches for the inherent 4T1-SCA1^+^ population, rather than converting SCA1^–^ cells into SCA1^+^ cells. In addition, *Osmr*, *Il6*, *Il6ra*, and *Il6st* genes were all overexpressed in MR13 cells compared with parental 4T1 cells, while *Osm* expression was not altered (Figure 7C). To functionally validate the IL-6/JAK signaling pathway in SCA1^+^ population maintenance, we treated MR13 cells with ruxolitinib in vitro for 48 hours. The treatment significantly decreased the fraction of SCA1^+^ cells (Figure 7D), while cell proliferation (Figure 7E and Supplemental Figure 8E) and viability (Supplemental Figure 8, F and G) were not affected. Interestingly, in vitro pretreatment of MR13 cells with ruxolitinib for 3 days completely abolished their lung metastatic capacity upon tail-vein injection (Figure 7, F–H). Together, these data indicate that MR13 cells sustain the metastatic SCA1^+^ population through cell-autonomous activation of the IL-6/JAK signaling pathway.

### Tu-Gr1^+^CD11b^+^–invoked tumor cell signature predicts shorter OS and RFS in breast cancer patients.

**Figure 8:**
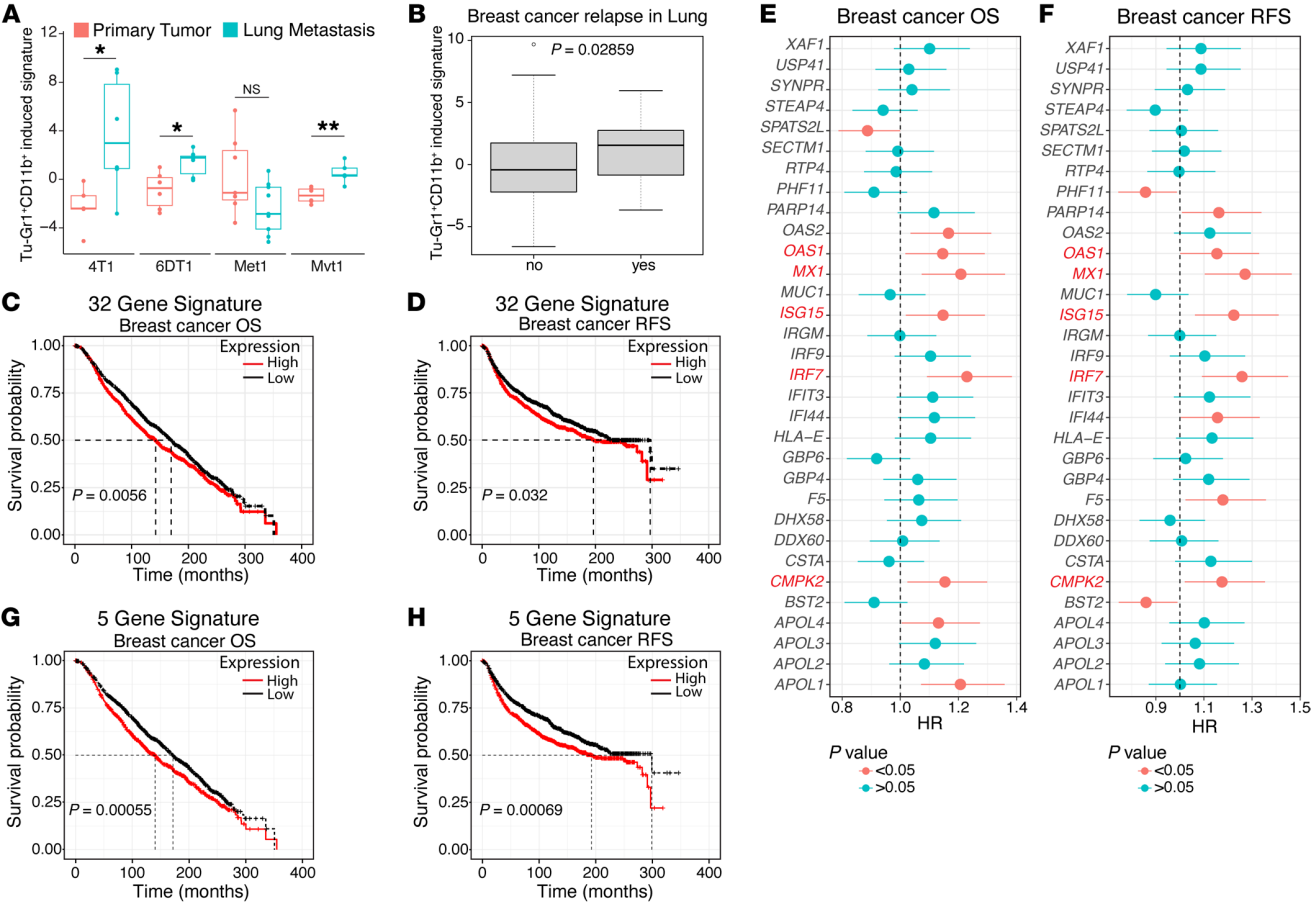
Tu-Gr1^+^CD11b^+^–induced tumor cell signature predicts worse outcome in breast cancer patients. (**A**) Box plot showing the expression of Tu-Gr1^+^CD11b^+^–induced 4T1 cell signature in PT and lung metastasis in the 4T1, 6DT1, Mvt1, and Met1 murine metastatic breast cancer models, extracted from the Ross data set (39). The box extended from 25th to 75th percentile, with the median indicated as a line within the box. The whiskers shown are 1.5 times interquartile ranges. *P* values were calculated using unpaired 2-tailed Student’s *t* test. (**B**) Expression of human orthologue of Tu-Gr1^+^CD11b^+^–induced signature in breast cancer patients with (yes) or without (no) metastatic relapse to the lung in the NKI295 cohort (58). (**C** and **D**) Kaplan-Meier curves showing OS (**C**) or RFS (**D**) for breast cancer patients according to high or low expression of an orthologue 32 gene signature, based on the Tu-Gr1^+^CD11b^+^–induced 4T1 cell signature in the METABRIC data sets (59). (**E** and **F**) Forest plots showing Cox’s proportional hazard regression (HR) for OS (**E**) and RFS (**F**) of the individual 32 orthologues of the Tu-Gr1^+^CD11b^+^–induced signature, based on gene expression in tumor samples of the METABRIC data set. (**G** and **H**) Kaplan-Meier curves showing OS (**G**) and RFS (**H**) according to the minimal 5-gene orthologue gene signature expression in the METABRIC data sets. *P* values were calculated using unpaired 2-tailed Student’s *t* test (**A** and **B**) or log-rank test (**C**, **D**, **G** and **H**). *P < 0.05; **P < 0.01.

To evaluate the clinical relevance of the crosstalk between Tu-Gr1^+^CD11b^+^ and tumor cells, we tested to determine whether the Tu-Gr1^+^CD11b^+^–induced 4T1 signature (top 50 DEGs) could predict cancer progression in other murine models and patients. First, we examined the signature expression in the PT and lung metastasis from different mouse models using the Ross et al. data set (39). The signature was significantly elevated in lung metastasis in 4T1, 6DT1, and Mvt1 breast cancer models, while in the Met1 model, it was unaltered (Figure 8A). Next, we examined the NKI295 cohort (58) consisting of expression data of invasive breast carcinoma from 295 women curated with site-specific metastasis data. Higher expression of the human orthologue of the murine signature predicted metastatic relapse to the lung (Figure 8B). Further, we interrogated the METABRIC data set comprising expression data from over 2,000 breast cancer patients (59) with the Tu-Gr1^+^CD11b^+^–induced signature. Thirty-two human orthologue genes (Supplemental Table 3) of the murine 50 gene signature were present in the METABRIC data set. Patients with higher expression levels of the orthologue signature had shorter OS (*P* = 0.0056) and RFS (*P* = 0.032) for all patients. Median OS and RFS values of patients with high versus low signature were 142.4 versus 169.6 and 196.4 versus 296.7 months, respectively (Figure 8, C and D). When interrogating breast cancer subtypes, higher signature expression was associated with a significantly reduced OS in ER^+^, PR^+^, and HER2^–^ breast cancer patients (Supplemental Figure 9, A–F). Notably, *OSM* expression levels positively correlated with the signature (Supplemental Figure 9G), suggesting that OSM does contribute to altered signature expression in breast cancer patients. Of the 32 genes, 5 (*MX1*, *IRF7*, *OAS1*, *CMPK2*, *ISG15*) were discriminant for a shorter OS and RFS (Figure 8, E and F), and when combined they showed enhanced predictive power for OS (*P* = 0.00055) and RFS (*P* = 0.00069) (Figure 8, G and H).

## Discussion

Metastatic disease and therapy resistance are the leading causes of breast cancer mortality, calling for alternative approaches to effectively prevent and treat metastasis and overcome therapy resistance. It has been proposed that CSCs present in the PT are responsible for tumor persistence, metastasis, and therapy resistance (54, 60). CSC features can be intrinsic or plastic and, importantly, can be modulated by cues from the TME (61–63).

Using the murine 4T1 and D2A1 experimental models, supported by transcriptomic analyses, we show in this study that SCA1^+^ populations with CSC features can exist under 3 different conditions: (a) inherently to the tumor cell lines; (b) induced upon tumor cell exposure to Tu-Gr1^+^CD11b^+^ cells; and (c) selected from tumor cells following treatment with chemotherapy. All these populations exhibit higher metastatic abilities compared with their counterpart controls. The distinct gene expression signatures of these 3 SCA1^+^ populations suggest a remarkable plasticity of these cells. GSEA analysis demonstrates that the Tu-Gr1^+^CD11b^+^–induced SCA1^+^ population is likely converted (twisted) from the SCA1^–^ population, while the SCA1^+^ population surviving chemotherapy (MR13) appears to be enriched (selected) from the inherent SCA1^+^ population. This plasticity is further supported by comparing single-cell gene expression of murine 4T1 and human MCF-7 breast cancer cells before and after in vivo growth. Also, Gong et al. (64) have reported that sorted SCA1^–^ 4T1 cells could be transiently transformed into a SCA1^+^ population by radiotherapy. An analogous observation was reported in colorectal cancer, where selective ablation of LGR5^+^ CSCs in organoids leads to initial tumor regression, followed by regrowth driven by LGR5^+^ CSCs reemerging from the LGR5^–^ population (65). Our work underscores the crucial observation that highly plastic CSC populations can be rapidly induced by tumor-educated granulocytic cells, giving rise to heterogeneous and expandable cell populations with high metastatic potential. Lineage-tracing experiments combined with time-course scRNA-Seq and spatial analyses within the PT will be necessary to characterize the detailed origin, development, function, and fate of the induced SCA1^+^ population during cancer progression.

Recruitment and education of Gr1^+^CD11b^+^ cells in the TME, particularly through the chemokines CCL2, CXCL1, and CXCL2 or IL-33, are considered critical steps for their contribution to tumor progression and metastasis (66–69). Here, we show that upon recruitment, these cells are endowed with an enhanced ability to induce metastatic SCA1^+^ cells via a positive amplification mechanism: recruited Gr1^+^CD11b^+^ cells are tumor educated to elevate IL-6/OSM expression, which, in turn, induces SCA1^+^ cells with enhanced capacity to form metastases from low metastatic SCA1^–^ cells. In addition, SCA1^+^ cells are more effective in recruiting Gr1^+^CD11b^+^ and educating them to elevate IL-6/OSM expression, creating a positive prometastatic reinforcement loop. Recently, it was reported that neutrophils escorting blood circulating tumor cells (CTCs) expand the metastatic potential of CTCs (70). While this effect was attributed to the promotion of cell-cycle progression of CTCs through direct contact with the neutrophils, in light of our findings, one may also consider the possibility that neutrophils may promote the expansion of a CSC-like phenotype with higher metastatic capacity while clustered with CTC traveling in the circulation. OSM was reported to be expressed by neutrophils cocultured with breast cancer cells and to promote phenotypic changes associated with mesenchymal and stem cell–like differentiation in breast cancer (37, 71). These findings further reinforce the notion that boosting the OSM expression in Gr1^+^CD11b^+^ cells is part of their educating program prompted by the tumor to create a paracrine signaling loop.

On the other side, OSM has also been shown to remodel macrophages and fibroblasts of the TME (34, 37). Araujo et al. recently reported that OSM derived from tumor-infiltrating myeloid cells reprograms fibroblasts to secrete VEGF and the chemokines CXCL1 and CXCL16, resulting in enhanced myeloid cell recruitment and breast cancer progression (34). IL-6 has been implicated in numerous protumoral effects in breast cancer, including promotion of metastasis by hijacking ER transcriptional program (72), CSC maintenance and chemoresistance (73), and recruitment of myeloid-derived suppressor cells (MDSCs) (74). In the present study, we extend these observations by demonstrating that Tu-Gr1^+^CD11b^+^–derived IL-6 and OSM twist cancer cell plasticity by promoting a rapid but reversible conversion of SCA1^–^ cells into more metastatic SCA1^+^ cells.

Exploring tumor-derived factors that educate the Gr1^+^CD11b^+^ cells within the TME to foster tumor plasticity represents a captivating avenue of investigation. Our analysis revealed a noteworthy upregulation of *Tgf*β*3* and *Tslp* in both SCA1^+^ and MR13 cells compared with the relative controls, suggesting their potential role as mediators of granulocytic cell education in accordance with cell-cell interaction analysis. TGF-β3 and TSLP were reported to be expressed in breast cancer and associated with both pro- and antitumor activities (75–78). Additional studies are warranted to delve deeper into the specific mechanisms through which TGF-β3, TSLP, and possibly other tumor-derived factors may contribute to myeloid cell education and elevated IL-6 and OSM expression.

Importantly, we demonstrate that a human orthologue of the murine Tu-Gr1^+^CD11b^+^–invoked tumor cell signature can predict relapse with lung metastasis and significantly shorter OS and RFS in breast cancer patients. Specifically, high orthologue signature predicts shorter OS, and to a lesser extent shorter RFS, for patients with ER^+^, HER2^–^, and PR^+^ cancers. These findings strengthen the clinical importance of the observed crosstalk between Tu-Gr1^+^CD11b^+^ and tumor cells. Strikingly, the 5 genes that contribute to the discriminatory power of the signature are genes related to native or viral immunity or regulated by interferon. While expression of interferons and interferon-response genes in breast cancer has been mainly associated with tumor suppression and improved survival (79), there is evidence also correlating interferon responses with tumor promotion, therapy resistance, and reduced survival (80). As JAK/STAT signaling is activated by both IFN and OSM/IL-6 receptors, it is conceivable that OSM/IL-6 only activates a subset of the IFN-induced genes with tumor-promoting activity, as is the case for Mx1 (81). Consistent with our findings, JAK/STAT signaling has recently been shown to initiate lineage plasticity in prostate cancer as well as to promote lineage plasticity–driven targeted therapy resistance in a stem-like subpopulation of prostate cancer (82, 83). On the other hand, Aouad et al. showed that epithelial-mesenchymal plasticity is essential for the generation of a dormant cell state of ER^+^ breast cancer during progression and the activation of IL-6/JAK/STAT signaling triggers tumor cell awakening and recurrence (50).

**Figure 9:**
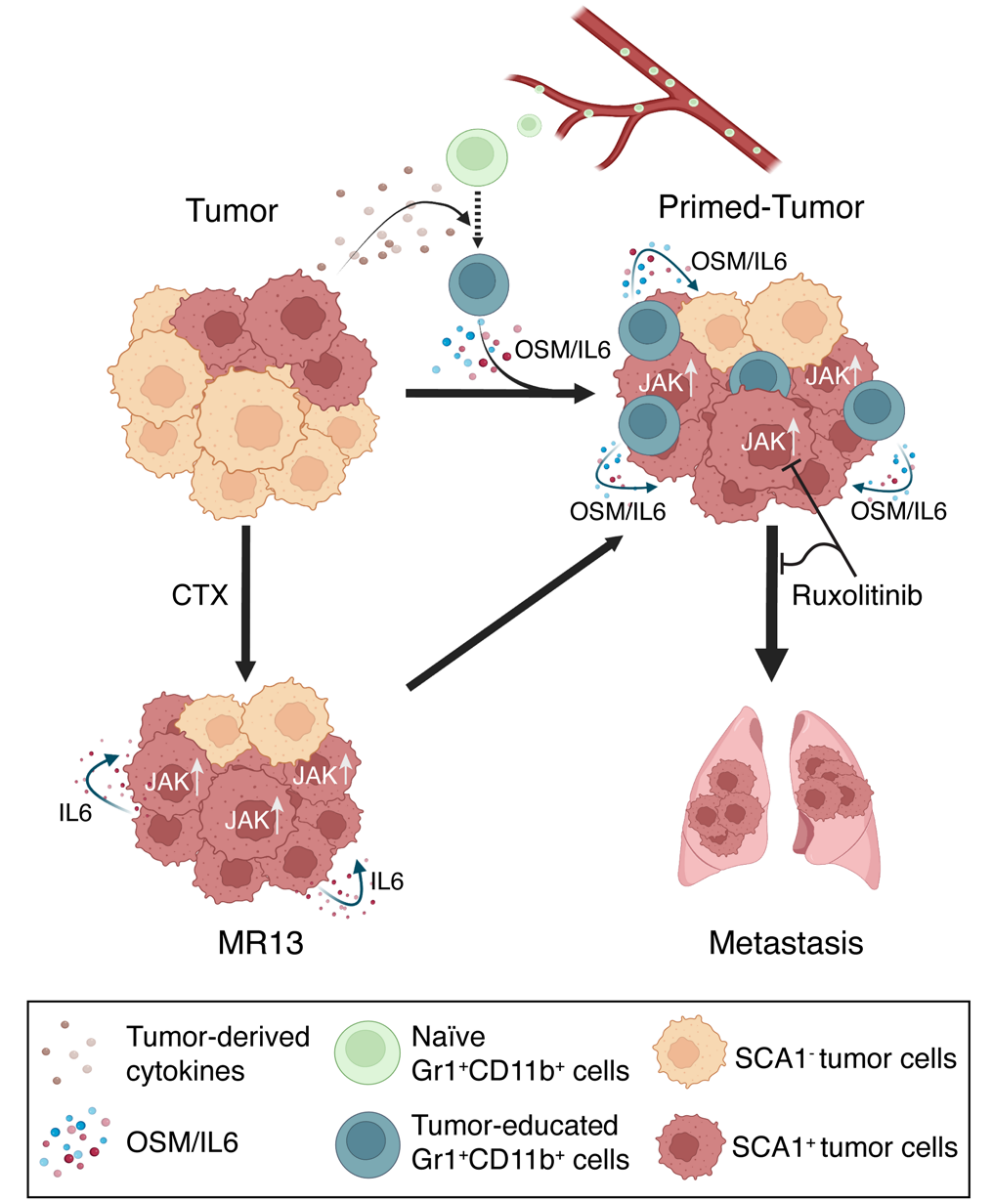
Schematic of the proposed model for cancer cell plasticity modulated by OSM/IL-6 during tumor progression and chemotherapy. Parental tumor cells contain a small fraction of highly metastatic SCA1^+^ population. During tumor progression, naive Gr1^+^CD11b^+^ cells are recruited to the TME and educated into Tu-Gr1^+^CD11b^+^ by tumor-derived factors. In turn, Tu-Gr1^+^CD11b^+^ cells secrete OSM and IL-6 to convert the SCA1^–^ population into a highly metastatic SCA1^+^ population. Chemotherapy (CTX) enriches for SCA1^+^ population due to its intrinsic resistance against cytotoxic treatment. Resistant cells express IL-6 to maintain the high portion of SCA1^+^ population with high metastatic ability. JAK inhibitor ruxolitinib suppresses the conversion to SCA1^+^ population and metastasis.

In conclusion, our findings unveil a clinically relevant model of breast cancer cell plasticity (Figure 9). We demonstrate that tumor-educated granulocytic leukocytes can manipulate tumor cell plasticity through OSM/IL-6/JAK signaling, promoting the emergence of a highly metastatic SCA1^+^ CSC population that is transcriptionally distinct from the endogenous SCA1^+^ CSC population. We also show that this process can be hijacked by tumor cells that survived chemotherapy and acquired high metastatic capacity through cell-autonomous mechanisms. Notably, a short in vitro treatment of MR13 cells with the JAK inhibitor ruxolitinib profoundly suppressed their metastatic capacity in vivo. However, a targeted inhibition of JAK using ruxolitinib in adjuvant or therapeutic settings might not be practicable, as the JAK pathway plays pivotal roles in both anti- and protumor immune responses (84). This concern is supported by a recent report showing that in vivo treatment with ruxolitinib did not suppress tumor growth and progression due to the abrogation of antitumor CD8^+^ T cell function through ruxolitinib-mediated inhibition of JAK1/2-dependent IFNGR1 signaling in T cells (85). Consistently, clinical trials with ruxolitinib for advanced or metastatic breast cancer failed to demonstrate clinical benefits (86–88). As depletion of OSM and IL-6 from conditioned medium of Tu-Gr1^+^CD11b^+^ is sufficient to suppress in vivo metastasis of in vitro–cocultured tumor cells, OMS/IL-6 inhibition may be considered as an alternative adjuvant strategy to prevent progression to metastases, with potential clinical benefit for patients. Patient stratification based on the granulocytic cell–induced signature may be valuable in identifying individuals likely to benefit from this therapeutic approach.

## Methods

### Sex as a biological variable.

Our study exclusively examined female mice because the vast majority of breast cancer occurs in females and hence, results in female mice are clinically relevant to human females.

### Tumor models.

4T1, MR13, sorted 4T1-SCA1^+^, and 4T1-SCA1^–^ cells (5 × 10^4^ cells in 50 μl PBS/10% of 8.1 mg/ml MG) were injected in the fourth right mammary glands of BALB/c female mice (Charles River). Prior to surgery, ketamine (1.5 mg/kg) and xylazine (150 mg/kg) (both from Graeub) were injected i.p. to anesthetize the animals. Immune cell populations were analyzed at different time points after tumor cell injection. Tumor length and width were measured twice a week with calipers and used to calculate tumor volume by the following equation: volume = (length × width^2^) × π/6. Tumors were collected and weighed at necropsy. For the i.v. injections, 2 × 10^5^ sorted 4T1-SCA1^+^ and 4T1-SCA1^–^ tumor cells resuspended in a volume of 50 μl of PBS were injected into the mouse tail veins. Lung metastases were quantified 10 days after injection. At the indicated time points, mice were sacrificed according to defined ethical criteria and were killed by CO_2_ inhalation followed by neck dislocation or terminal bleeding.

### Cell culture.

The 4T1 murine breast cancer cell line was provided by Fred R. Miller (Michigan Cancer Foundation, Detroit, Michigan, USA). 4T1 cells were cultured in high-glucose DMEM supplemented with 10% heat-inactivated FBS, 1% penicillin-streptomycin (P/S) (Gibco, Thermo Fisher Scientific), and 1% nonessential amino acids (Gibco, Thermo Fisher Scientific). The D2A1 murine breast cancer cell line was provided by Jonathan Sleeman (Medical Faculty at Heidelberg University, Mannheim, Germany). D2A1 cells were cultured in high-glucose DMEM supplemented with 10% heat-inactivated FBS, 1% P/S.

### Reagents and chemicals.

Growth factor reduced Matrigel Matrix (MG) was obtained from BD Biosciences. Collagenase I was purchased from Worthington and DNAse I from Roche. BSA, crystal violet (CV), and paraformaldehyde (PFA) were obtained from Sigma-Aldrich. Dox and MTX were provided by Dr. Khalil Zaman from the Department of Oncology, University Hospital, University of Lausanne, Lausanne, Switzerland. Ruxolitinib (JAK inhibitor, catalog 7064) was purchased from Bio-Techne and used at 5 μg/ml. The anti-mouse OSM (R&D systems Clone: P53347), anti-mouse IL-6 (BioxCell, Clone MP5-20F3) were used at 1 μg/ml. The recombinant mouse OSM and IL-6 (BioLegend, catalog 762802 and 575702, respectively) were both used at 10 ng/ml.

### Antibodies.

The following anti-mouse antibodies were used: anti-CD16/CD32 Fc blocking antibody (clone 2.4G2) and anti–CD45-PE (clone 30-F11) (BD Biosciences); anti–CD24-FITC (clone M1/69), anti–SCA1-APC (clone D7) anti–Gr1-eFlour450 (clone RB6-8C5), anti–Ly6G-APC (clone 1A8-Ly6g), anti-F4/80 PerCP/Cy5 (clone BM8), anti–CD11b-PE-Cy7 (clone M1/70), anti–CD11c-APC-eFluor780 (clone N418), anti–CD4-FITC (clone 6K1.5), anti–CD8-PE (clone 53-6.7), and anti–B220-APC (clone RA3-6B2) (eBioscience); anti–CD61-Alexa Fluor 647 (clone 2C9.62[HMβ3-1]), anti–Ly6C FITC (clone HK1.4), anti–CD49b-eFluor450 (clone DX5), annexin V–APC (clone B217656) (BioLegend); and propidium iodide–PerCP (clone V13245) (Life Technologies).

### Coculture of Gr1^+^CD11b^+^ cells sorted from tumors or spleen of 4T1 tumor-bearing mice.

Gr1^+^ cells were isolated from tumors or spleen at day 23 after injection (see *Magnetic cell sorting (MACS)* in Supplemental Methods). Afterwards, 1.5 × 10^5^ 4T1 or sorted 4T1-SCA1^–^ cells (bottom well) were cocultured in 6-well Transwell plates (0.4 μm, Nunc, ThermoFisher Scientific) with Gr1^+^CD11b^+^ sorted cells (top well) at different ratios. After 48 hours of coculture, cells were analyzed for SCA1 expression and the primed cells (4T1 or 4T1-SCA1^–^) were used for further in vivo or in vitro experiments.

### Flow cytometry analysis on tissue samples and sorting.

Mice were sacrificed at different time points for blood and tumor collection. Tumors were cut in small pieces with scissors, washed, and digested in serum-free medium supplemented with collagenase I and DNAse I (Roche). The mixture was incubated at 37°C for 45 minutes on a shaking platform. Subsequently, serum-supplemented medium was added to neutralize the enzymatic reaction and the tissue suspensions were filtered through 100 μm and 70 μm sterile nylon gauzes. Upon centrifugation (5 minutes at 400 g), pellets were recovered and red blood cells lysed with ACK buffer (BioLegend). The staining procedure and events acquisition by flow cytometry (FACSCalibur, BD Biosciences or MACSQuant flow cytometer from Miltenyi Biotec) were done as previously described (89). Cell sorting was done by FACSAria Fusion (BD Biosciences). Data were analyzed by FlowJo, version 10.0.7 (Tree Star Inc.).

### Statistics.

Statistical analyses were performed with Prism 7.0 (GraphPad Software) or R package ggpubr (version 0.4.0) (90). *P* values of less than 0.05 were considered significant. Statistical comparisons were performed using 2-tailed, unpaired Student’s *t* test or paired Student’s *t* test as indicated for comparing 2 different groups. For more than 2 groups, unpaired 2-tailed Student’s *t* test with Holm’s correction, 1-way ANOVA with Bonferroni’s post test, or 2-way ANOVA with Tukey’s or Dunnett’s multiple comparison was used as indicated.

### Study approval.

All animal procedures were performed in accordance with Swiss legislation on animal experimentation and approved by the Cantonal Veterinary Service of the Cantons Vaud and Fribourg for experiments in Lausanne and Fribourg (VD_1486.2; 2011_33_FR; 2014_58_FR; 2017_34_FR; 2021-29-FR).

### Data availability.

The transcriptomic data generated by this study have been deposited in the NCBI’s Gene Expression Omnibus database (GEO GSE215925). The code used for the analyses is open source and available through R packages as described in Supplemental Methods. Values for all data points in graphs are reported in the Supporting Data Values file.

## Author contributions

SP, QL, and CR conceived the study. SP, NF, QL, and CR developed experimental and analytical methods and protocols. AK, RM, NF, and QL established the pipeline for human transcriptomics data analysis. SP, MB, AY, AK, and QL repeated experiments and confirmed results. SP, MB, AY, AK, RM, and QL analyzed the data. SP, MB, AY, AK, RM, LH, ND, QL, and CR prepared the data for formal analysis. SP, MB, AK, RM, QL, and CR prepared the figures. SP, MB, AY, AK, RM, OC, ND, LG, and GL provided resources. SP, AK, YTH, GL, ND, QL, and CR reviewed and edited the manuscript. SP, AK, RM, ND, QL, and CR revised the manuscript. SP, AY, QL, and CR supervised the project. QL and CR performed project administration. CR acquired funding.

## Supplementary Material

Supplemental data

Supplemental table 1

Supplemental table 2

Supplemental table 3

Supporting data values

## Version 1 01/18/2024

In-Press Preview

## Version 2 03/15/2024

Electronic publication
