# Supplementary material for: Tumor-educated Gr1+CD11b+ cells drive breast cancer metastasis via OSM/IL-6/JAK–induced cancer cell plasticity
Source: J Clin Invest. 2024 Jan 18;134(6):e166847. doi: 10.1172/JCI166847 (PMC10940099; doi:10.1172/JCI166847)
Supplement: Supplemental data [file jci-134-166847-s190.pdf]

Supplemental Information

Supplemental Methods

Supplemental Figures 1-9

Supplemental Tables 1-3

Supplemental Methods

Magnetic cell sorting (MACS). MACS separators were used for positive and negative cell selections based on manufacturer's instructions. Briefly, cells were counted and resuspend in 500 µl of MACS buffer with 10 µl of fluorescent coupled antibody of interest (APC-conjugated anti-SCA1 and PE-conjugated anti-Gr1) per 10^7 cells added. Cells were incubated for 30 minutes in the dark at 4°C and then washed with MACS buffer. After centrifugation and resuspension in 80 µl of MACS buffer per 10^7 cells, 20 µl of anti- APC-conjugated magnetic beads (Miltenyi Biotec) per 10^7 cells was added. After a 20 minutes incubation in the dark at 4°C, cells were washed with MACS buffer. After the centrifugation they were resuspended in MACS buffer and the magnetic separation was performed using LS MACS column for positive selection and LD MACS column for negative selection. The purity of positive subpopulation was up to >90% and <99% for the negative subpopulation.

In vitro cell proliferation assay. Cells were collected and seeded in tissue culture 96-wells-plates (Costar) at 3000 cells/well. Cells were grown in complete medium for 24, 36, 72 and 96 hours. At each time point cells were washed once with PBS, then fixed with 4% PFA and stained with 0.5% crystal violet solution for 0.5 hours. The stained cells were gently washed with deionized water to remove the extra dye and air-dried overnight at room temperature. After solubilizing the dye with crystal violet eluting buffer (70% ethanol and 1% acetic acid), cell viability was assessed by reading the absorbance at 595 nm wavelength in a multiwell plate reader (Modulus II microplate reader, Turner Biosystems). Results were analyzed by Prism (Graph pad software, Inc.) expressed as mean values of optical density (OD) of octuplet determinations ± SEM.

In vitro cytotoxic assay. Tumor cells were plated at a concentration of 3000 cell/well into 96-wells plates. The following day, a series of concentrations of the different drugs were supplemented to the culture medium. Untreated control cells were kept in normal culture medium. Cell viability of each well was assessed with crystal violet staining 48 hours after treatment, as described above. Results were analyzed by Prism software by a non-linear regression analysis and expressed as relative cell viability compared with non-treated control. The 50% maximum inhibition concentrations (IC_{50}) were used to determine the drug-resistant ability of treated cells.

Histopathology. Tumors and lungs were harvested at the end of the experiments, fixed in formalin and embedded in paraffin. 5 µm thick serial sections were cut from the tissue blocks. 3-4 sections taken at 100 µm distance were stained with hematoxylin and eosin (H&E) and used to assess tumor morphology and quantify lung metastasis. Slides were scanned by Nanozoomer (Hamamatsu Photonics) and metastasis were counted manually using NDP.viewer2 software (Hamamatsu Photonics). Metastatic index was calculated by normalizing the metastasis number with the volume of primary tumor.

Mammosphere-forming assay 5000 cells/well were seeded in non-adhesive U bottom 96-wells plate in a semi-solid MEGM medium supplemented with 20 ng/ml EGF, and 20 ng/ml bFGF and heparin. Medium was gently replaced every 3 to 4 days. After 11 to 14 days culture, mammospheres of 50 to 150 µm diameter were detected under the microscope (bright field) and counted to quantify the sphere formation efficiency (SFE) as percentage of the initial number of seeded cells per well.

Cell proliferation analysis by EdU. EdU-Click detection Flow Cytometry Assay Kit (Sigma-Aldrich) were used to assess the cell proliferation status by flow cytometry. The cells were incubated with 10 μM EdU in complete medium for 2 hours at the end of experiment, and staining was performed according to manufacturer's instructions. The DNA content was measured by DAPI staining (Life Technology). Data acquisition was performed using Fortessa flow cytometer (BD Bioscience) and data analyzed by FlowJo v10.0.9 (tree Stat Inc).

OSM and IL6 measurement by ELISA. 1.5×10^5 4T1 cells were cultured for 48 hours in 6 well plates with Tu-Gr1$^+$CD11b$^+$ or Spl-Gr1$^+$CD11b$^+$ cells. Supernatants were collected and stored at -80°C. OSM and IL6 ELISA was performed according to manufacturer's instructions (R&D Systems and Invitrogen, respectively).

Apoptosis analysis. Cell apoptosis detection was performed using an APC-Annexin V Apoptosis Detection Kit (eBioscience) according to manufacturer's instructions. Data acquisition was performed using the Fortessa flow cytometer from BD Bioscience and data analyzed by FlowJo v10.0.9 (tree Stat Inc.).

Lentiviral constructs and gene silencing by shRNA. Short hairpin RNA (shRNA) sequences targeting *Sca1*, namely *shSca1*-120 (TRCN0000100120) and *shSca1*-597 (TRCN0000327597), as well as a non-targeting sequence (*shNT*, SHC001) in pLKO.1-puro lentiviral vectors were purchased from Sigma-Aldrich. Lentivirus production, infection and selection with puromycin (5 μg/ml) were performed as previously described (1). Gene knocking down efficiency in 4T1 cells were verified by Flow cytometry to assess the expression of SCA1 after stimulating with recombinant OSM for 48 hours.

Real-time reverse transcription qPCR and primers. Changes in mRNA expression levels were determined by semi-quantitative real-time qPCR. RNA was isolated using RNeasy kit from QIAGEN according to manufacturer's instructions. From each sample, 1 µg RNA was retro-transcribed using SuperScript II Reverse Transcriptase kit (Life Technologies – Invitrogen), according to manufacturer's instructions. The reactions were performed in a StepOnePlus™ thermocycler (Applied Biosystems, Life Technologies – Invitrogen) using the KapaSYBR® FAST SYBR Green Master Mix (Kapa Biosystems). Each reaction was performed in triplicates and values were normalized to murine 36B4 housekeeping gene. The comparative C_t method was used to calculate the difference of gene expression between samples. The following murine-specific primers (Microsynth AG) were used:
36b4 (F:5'-GTGTGTCTGCAGATCGGGTAC-3',R:5'-CAGATGGATCAGCCAGGAAG-3'), *Osm* (F:5'-ATGCAGACACGGCTTCTAAGA-3', R:5'- TTGGAGCAGCCACGATTG G-3'), *Il6*: (F:5'- TACCACTTCACAAGTCGGAGGC-3', R:5'- CTGCAAGTGCATCAT CGTTGTTC-3').

Bulk RNA sequencing and data analysis. Four independent sorts of 4T1-SCA1$^+$ and 4T1-SCA1$^-$ cells (as described in the magnetic cell sorting section) or four independent 4T1 cells primed with Tu- Gr1$^+$CD11b$^+$ or Spl-Gr1$^+$CD11b$^+$ were prepared. RNAs of these cells were isolated using the NucleoSpin RNA protocol of Macherey-Nagel (as described in real-time (RT) qPCR and primers section). Samples were normalized for 1 µg RNA in a volume of 20 µl, sequenced on the NextSeq500 sequencer using the NextSeq 500/550 HT reagent v2 kit (Illumina) at the Swiss Integrative Center for Human Health (SICHH) in Fribourg, or the Lausanne Genomics Technologies Facility (GTF, UNIL) in Lausanne, Switzerland. For data analysis, all sequencing reads were processed for quality control, removal of low quality reads, adaptor sequence and ribosomal RNA by fastqc(0.11.8) (2), multiqc (1.9) (3),

Trimmomatic (0.39) (4) and SortMeRNA(2.1) (5) accordingly. The filtered reads were mapped to the reference genome (mm10) using htseq-count (0.6.1) (6) or Salmon (0.99.0) (7). The normalization of the read counts and the analysis of the differential expression between the groups of samples were performed with in R(v4.1.3), a free software environment available at https://www.r-project.org/ using packages DESeq2 (v3.15) (8). Pathway enrichment analysis was performed using packages GSVA(1.42.0) (9) and GSEABase(1.56.0) (10) with the input of DESeq2 normalized count numbers using ssgsea method comparing the Hallmark genesets from MSigDB (v7.4.1) (11) with default settings. The significant altered pathways were determined by computing moderated t-statistics and false discovery rates with the limma(3.50.3)(12) for pairwise comparison. The heatmaps were produced with R package pheatmap(1.0.12)(13) with default settings while pathway hierarchy clustering was performed by similarity based on Euclidean distance and the ward aggregation algorithm. The Sca-1 Positive and Sca-1 Negative signatures were extracted from the top 200 most upregulated or downregulated genes in 4T1- SCA1^{+} and 4T1- SCA1^{-} RNAseq data, respectively, with the threshold of adjusted p-value <0.05, fold change >1.5 or <-1.5 and average normalized count number >20. The Tu-Gr1^{+}CD11b^{+}-induced signature was extracted by comparing gene expression between Tu-Gr1^{+}CD11b^{+} and Spl-Gr1^{+}CD11b^{+}-educated 4T1 cells with adjusted p value <0.05, fold change >2, and the top 50 genes were selected. For Venn diagram, the genes fulfilling the threshold of adjusted p-value < 0.05, fold change > 1.5 or < -1.5 and average normalized count number >20 are compared. The figures were produced with R package venn (1.10) (14) . Further analysis and figures generation were performed in R using packages tidyverse (1.3.1) (15), ggplot2(3.3.6) (16), circlize(0.4.15) (17), biomaRt(2.50.3) (18, 19), RColorBrewer (1.1-3) (20), clusterProfiler (4.2.2) (21, 22), enrichplot (1.14.2) (23), ggpubr (v0.4.0) (24) ggbreak (0.1.0) (24).

Microarray hybridization and data analysis Experiments were performed as previously described (25). Briefly, triplicates wells of cultured 4T1 and MR13 cells were used for RNA extraction using RNeasy kit (QIAGEN). Probe synthesis and GeneChip Mouse Gene Exon 1.0 ST Array (Affymetrix Ltd) hybridization were performed at the GTF, UNIL, Lausanne, Switzerland. Microarray analyses were carried out with R. After quantification of gene expression with robust multi-array normalization (19) using the BioConductor package Affy, (http://www.bioconductor.org/) significance of differential gene expression was determined by computing moderated t-statistics and false discovery rates with the limma package (12). Annotation was based on the genome version NCBI Build 36 (Feb. 2006). The obtained p-values were corrected for multiple testing by calculating estimated false discovery rates (FDR) using the method of Benjamini-Hochberg. Heatmaps were produced by color-coding gene-wise standardized log gene expression levels (mean zero standard deviation one). Probe-sets were shown hierarchically clustered by similarity based on Euclidean distance and the ward aggregation algorithm.

Public RNAseq data analysis The RPKM normalized gene expression data in Ross dataset (GSE150928) from multiple murine models of breast cancer metastasis was obtained from Gene Expression Omnibus (GEO) in the NCBI data repository. The expression of Tu-Gr1^{+}CD11b^{+}-induced signature was calculated using the hack_sig function within R package hacksig (26) employing the zscore method with default settings. Outliers in each group were identified based on being beyond 2 times the Interquartile Range (IQR) from upper and lower quartiles. The data were analyzed and plot with R package tidyverse(1.3.1) (15), ggplot2 (3.3.6) (16) and ggpubr (v0.4.0) (27). The single-cell RNAseq data in Sebastian dataset (28) was obtained from Dryad data repository (https://doi.org/10.6071/M3238R). The data were analyzed with R package Seurat (3.0) (29). The tumor and different myeloid cell populations

were extracted for cell-cell interaction analysis using CellPhoneDB (2.0) (30). The identified interaction pairs were extracted and plot using circlize (0.4.15) (17) and ComplexHeatmap (2.11.2) (31). Integrin genes were excluded for better visualization.The ligands and receptors annotated in the circular plot were compiled from databases in CellTalkDB(32), SingleCellSignalR (33). To investigate the tumor cell dynamics, datasets for 4T1 (GSE158844 and GSM3502134) and MCF-7 (GSM4681765 and GSM5904917) were obtained from GEO. The data were filtered and normalized separately before merging with IntegrateData function included in Seurat. Cell cycle regression was performed according to the standard protocol of Seurat. For MCF-7, due the huge difference of the sample size, 2000 cells were randomly selected from GSM4681765 data, and then merged with GSM5904917. Single-cell trajectories analysis was performed with monocle 3 (34–37) with default settings and the root and start point were selected manually for pseudo time calculation. The GSEA analysis of selected clusters was performed with R package fgsea (38) and the gene rank was calculated with Wilcoxon rank sum test and auROC analysis using wilcoxauc function included in presto package.

Clinical data analysis. To validate our finding in clinical data, the human orthologs of murine Tu-Gr1$^+$CD11b$^+$-induced signature genes were used. Conversion from murine to human gene symbols and Entrez IDs was performed with the biomaRt package (2.46.3) (18, 19), using the reference mart https://dec2021.archive.ensembl.org. NKI295 data was retrieved from (39). Zscore normalization and signature assessment was performed using the hacksig package. Unpaired student's t-test was used to compare individuals with or without relapse disease in lung. Molecular Taxonomy of Breast Cancer International Consortium (METABRIC) breast cancer data was downloaded from cBioPortal (40–42) in August 2022, and expression data was log2 transformed. Expression values were stratified in two groups

by median values. Survival curves were generated using the ggsurvplot function from the survminer package (0.4.9) (27), and were compared between groups using a log-rank test. Survival curves were created using the survfit function from the survival package (3.2.11) (43). Cox proportional hazard regression model was performed through the coxph function of the same package.

References

1. Lorusso G, et al. Connexins orchestrate progression of breast cancer metastasis to the brain by promoting FAK activation. *Sci Transl Med*. 2022;14(661):eaax8933.

2. FASTQC. A quality control tool for high throughput sequence data | BibSonomy [Internet]. https://www.bibsonomy.org/bibtex/f230a919c34360709aa298734d63dca3. Accessed October 15, 2022.

3. Ewels P, et al. MultiQC: summarize analysis results for multiple tools and samples in a single report. *Bioinformatics*. 2016;32(19):3047–3048.

4. Bolger AM, Lohse M, Usadel B. Trimmomatic: a flexible trimmer for Illumina sequence data. *Bioinformatics*. 2014;30(15):2114–2120.

5. Kopylova E, Noé L, Touzet H. SortMeRNA: fast and accurate filtering of ribosomal RNAs in metatranscriptomic data. *Bioinforma Oxf Engl*. 2012;28(24):3211–3217.

6. Anders S, Pyl PT, Huber W. HTSeq—a Python framework to work with high-throughput sequencing data. *Bioinformatics*. 2015;31(2):166–169.

7. Patro R, et al. Salmon provides fast and bias-aware quantification of transcript expression. *Nat Methods*. 2017;14(4):417–419.

8. Love MI, Huber W, Anders S. Moderated estimation of fold change and dispersion for RNA-seq data with DESeq2. *Genome Biol*. 2014;15(12):550.

9. Hänzelmann S, Castelo R, Guinney J. GSVA: gene set variation analysis for microarray

and RNA-Seq data. *BMC Bioinformatics*. 2013;14(1):7.

10. Morgan M, Falcon S, Gentleman R. GSEABase: Gene set enrichment data structures and methods. R package version 1.58.0. 2022.

11. Subramanian A, et al. Gene set enrichment analysis: A knowledge-based approach for interpreting genome-wide expression profiles. *Proc Natl Acad Sci*. 2005;102(43):15545–15550.

12. Ritchie ME, et al. limma powers differential expression analyses for RNA-sequencing and microarray studies. *Nucleic Acids Res*. 2015;43(7):e47.

13. Kolde R. Pheatmap: pretty heatmaps. *R Package Version*. 2012;1(2):726.

14. Dusa, Adrian. Draw Venn Diagrams R package venn. 2016.

15. Wickham H, et al. Welcome to the Tidyverse. *J Open Source Softw*. 2019;4(43):1686.

16. Wickham H. *ggplot2*. New York, NY: Springer; 2009.

17. Gu Z, et al. circlize Implements and enhances circular visualization in R. *Bioinforma Oxf Engl*. 2014;30(19):2811–2812.

18. Durinck S, et al. BioMart and Bioconductor: a powerful link between biological databases and microarray data analysis. *Bioinforma Oxf Engl*. 2005;21(16):3439–3440.

19. Durinck S, et al. Mapping identifiers for the integration of genomic datasets with the R/Bioconductor package biomaRt. *Nat Protoc*. 2009;4(8):1184–1191.

20. Neuwirth E. RColorBrewer: ColorBrewer Palettes;5.

21. Wu T, et al. clusterProfiler 4.0: A universal enrichment tool for interpreting omics data. *Innov Camb Mass*. 2021;2(3):100141.

22. Yu G, et al. clusterProfiler: an R package for comparing biological themes among gene clusters. *Omics J Integr Biol*. 2012;16(5):284–287.

23. Yu G, Hu E. enrichplot: Visualization of Functional Enrichment Result. 2022. https://doi.org/10.18129/B9.bioc.enrichplot.

24. Xu S, et al. Use ggbreak to Effectively Utilize Plotting Space to Deal With Large Datasets and Outliers. *Front Genet*. 2021;12:774846.
25. Monnier Y, et al. CYR61 and alphaVbeta5 integrin cooperate to promote invasion and metastasis of tumors growing in preirradiated stroma. *Cancer Res*. 2008;68(18):7323–7331.
26. Carenzo A, et al. hacksig: a unified and tidy R framework to easily compute gene expression signature scores. *Bioinforma Oxf Engl*. 2022;38(10):2940–2942.
27. Kassambara A. ggpubr: “ggplot2” Based Publication Ready Plots. 2020. https://CRAN.R-project.org/package=ggpubr. Accessed October 15, 2022.
28. Sebastian A, et al. Single-Cell Transcriptomic Analysis of Tumor-Derived Fibroblasts and Normal Tissue-Resident Fibroblasts Reveals Fibroblast Heterogeneity in Breast Cancer. *Cancers*. 2020;12(5):1307.
29. Stuart T, et al. Comprehensive Integration of Single-Cell Data. *Cell*. 2019;177(7):1888-1902.e21.
30. Efremova M, et al. CellPhoneDB: inferring cell–cell communication from combined expression of multi-subunit ligand–receptor complexes. *Nat Protoc*. 2020;15(4):1484–1506.
31. Gu Z, Eils R, Schlesner M. Complex heatmaps reveal patterns and correlations in multidimensional genomic data. *Bioinforma Oxf Engl*. 2016;32(18):2847–2849.
32. Shao X, et al. CellTalkDB: a manually curated database of ligand–receptor interactions in humans and mice. *Brief Bioinform*. 2021;22(4):bbaa269.
33. Cabello-Aguilar S, et al. SingleCellSignalR: inference of intercellular networks from single-cell transcriptomics. *Nucleic Acids Res*. 2020;48(10):e55.
34. Trapnell C, et al. The dynamics and regulators of cell fate decisions are revealed by pseudotemporal ordering of single cells. *Nat Biotechnol*. 2014;32(4):381–386.
35. Qiu X, et al. Reversed graph embedding resolves complex single-cell trajectories. *Nat*

Methods. 2017;14(10):979–982.

36. Cao J, et al. The single-cell transcriptional landscape of mammalian organogenesis. *Nature*. 2019;566(7745):496–502.

37. McInnes L, Healy J, Melville J. UMAP: Uniform Manifold Approximation and Projection for Dimension Reduction [preprint]. 2020. http://arxiv.org/abs/1802.03426. Accessed October 21, 2022.

38. Korotkevich G, et al. Fast gene set enrichment analysis [preprint]. 2021;060012.

39. van de Vijver MJ, et al. A Gene-Expression Signature as a Predictor of Survival in Breast Cancer. *N Engl J Med*. 2002;347(25):1999–2009.

40. Gao W, et al. IL20RA signaling enhances stemness and promotes the formation of an immunosuppressive microenvironment in breast cancer. *Theranostics*. 2021;11(6):2564–2580.

41. Cerami E, et al. The cBio cancer genomics portal: an open platform for exploring multidimensional cancer genomics data. *Cancer Discov*. 2012;2(5):401–404.

42. Ramos M, et al. Multiomic Integration of Public Oncology Databases in Bioconductor. *JCO Clin Cancer Inform*. 2020;(4):958–971.

43. Therneau TM, Grambsch PM. *Modeling Survival Data: Extending the Cox Model*. New York, NY: Springer; 2000.

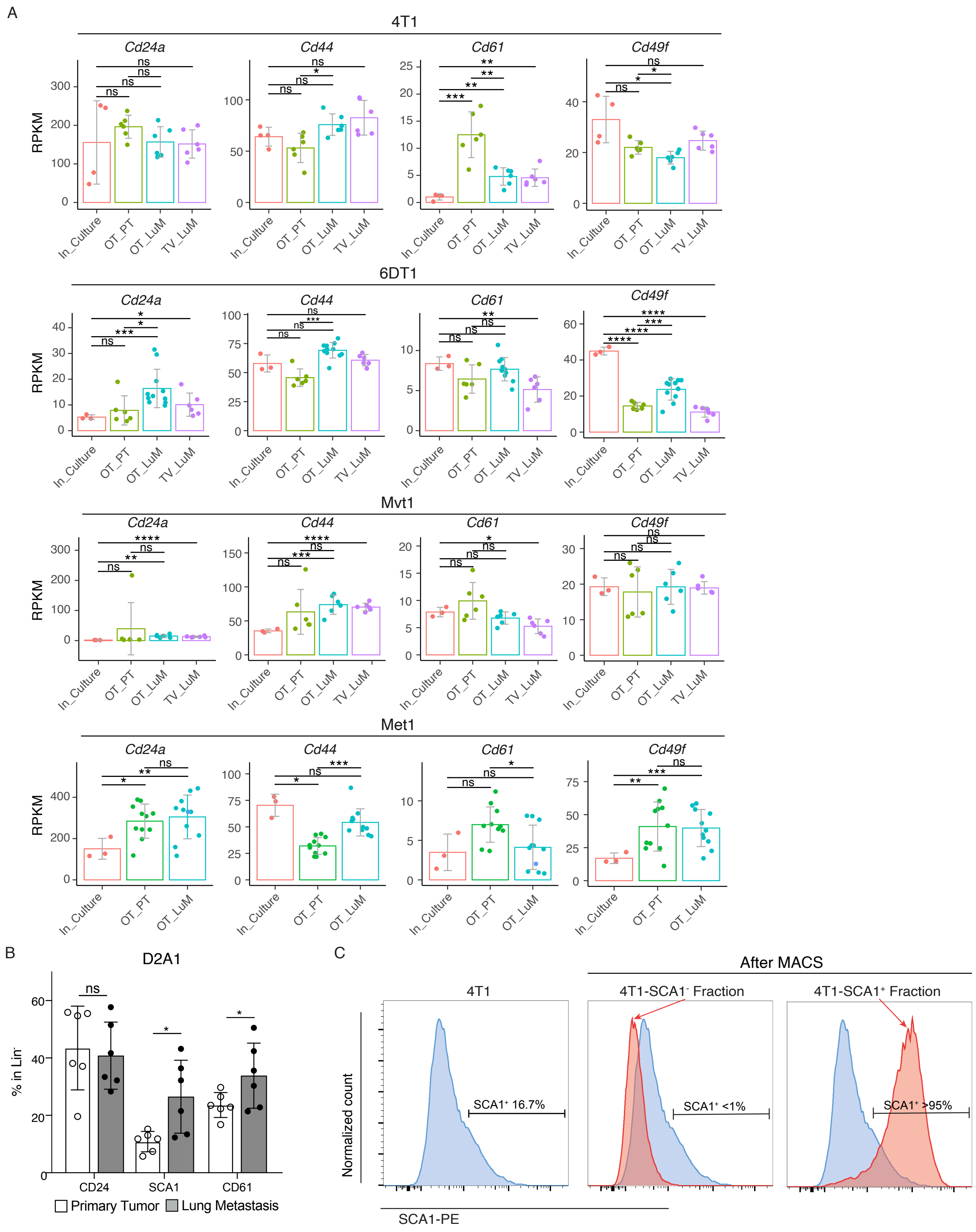

Supplemental Figure 1

Supplemental Figure 1. Stem cell markers expression in multiple metastatic breast cancer models

(**A**) Stem cell marker *Cd24a, Cd44, Cd61 and Cd49e* mRNA expression in 4T1, 6DT1, Mvt1 and Met1 metastatic murine breast cancer models extracted from Ross dataset. Data are presented as mean values of RPKM ± SD. *P* values were calculated using unpaired two-tailed student's *t* test with Holm correction.

(**B**) Frequency of CSC marker expression in primary tumors and lung metastases of D2A1 m.f.p injected mice D21-23 post injection. Results give the percentage of CD24, SCA1and CD61 positive cells gated in lineage negative cells ($CD45^-CD31^-TER119^-$). (n = 6/group). Data shown as mean ± SEM as representative of 3 independent experiments. *P* values were calculated using one-way ANOVA with Tukey multiple-comparison test.

(**C**) Histogram showing SCA1 expression distribution on parental 4T1 cells, MACS positively selected $SCA1^+$ cells and MACS negatively selected $SCA1^-$ cell.

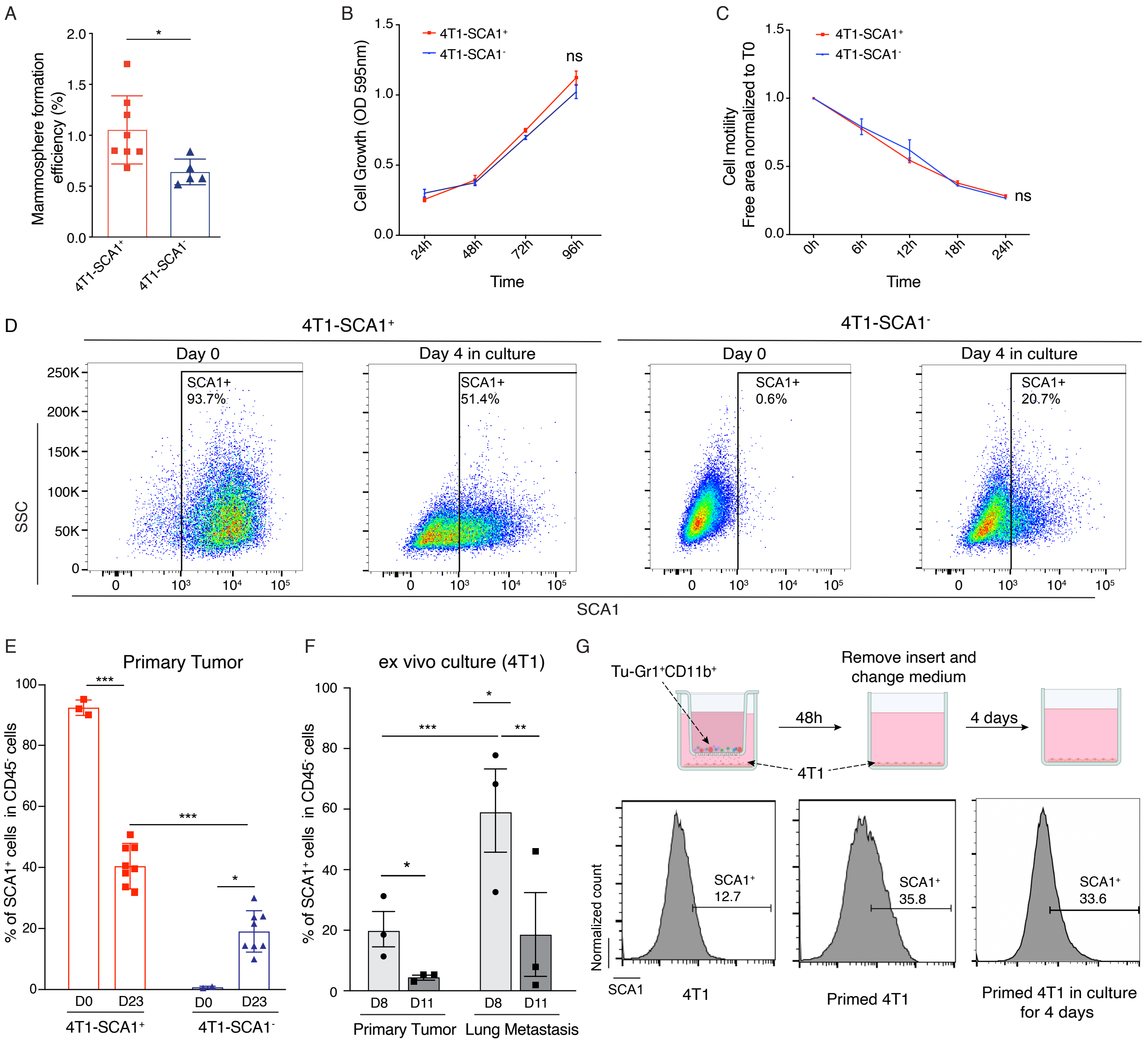

Supplemental Figure 2

Supplemental Figure 2. SCA1$^+$ tumor cells have stem cell-like features in vitro and show plasticity in vivo and in vitro

(**A**) Quantification of the mammosphere forming efficiency of 4T1-SCA1$^+$ and 4T1-SCA1$^-$ populations. (n=5-8/group).

(**B**) Cell proliferation curve of 4T1-SCA1$^+$ and 4T1-SCA1$^-$ in vitro MACS isolated tumor cells determined by crystal violet assay. The results represent optical density (OD) of the wells. (n=5-6/group).

(**C**) Cell motility of 4T1-SCA1$^+$ and 4T1-SCA1$^-$ sorted tumor cells determined by the scratch wound healing assay. (n=5-6/group). Results are given cell-free area relative to the initial wound area.

(**D**) The abundance of SCA1$^+$ population after MACS isolation from parental 4T1 cells. Day 0: immediately after positive sorting, the SCA1$^+$ population to accounts for 73.3% of total cells. After 4 days of in vitro culture the abundance of the SCA1$^+$ population decreased to 48.9%. Negatively sorted SCA1$^-$ population accounts for >99 % of total cells. After 4 days of in vitro culture 19.2% of the initially SCA1$^+$ cells were SCA1$^+$.

(**E**) Abundance of SCA1$^+$ population at the time of orthotopic injection of MACS isolated 4T1-SCA1$^+$ and 4T1-SCA1$^-$ cells into the mammary fat pad of BALB/c mice (D0) and in the derived primary tumors 23 days post injection (D23) as indicated. (n=3 technical replicates for D0 and n=8 for biological replicates D23).

(**F**) Abundancy of the SCA1$^+$ population in tumor cells recovered from primary tumors and lung metastases 21 days post orthotropic injection and further cultured for 8- and 11-days ex vivo as indicated. (n=3/group).

(**G**) SCA1 expression in parental 4T1 cells, 4T1 primed for 2 days with Tu-Gr1$^+$CD11b$^+$ cells and cultured for 4 additional days in the absence of Tu-Gr1$^+$CD11b$^+$ after priming. SCA1 expression was determined by flow cytometry.

Data shown as mean ± SEM as representative of 3 independent experiments. *P* values were calculated using unpaired two-tailed student's t test (**A**), two-way ANOVA with Tukey multiple-comparison test (**B**, **C**), or one-way ANOVA with Tukey multiple-comparison test (**E**, **F**).

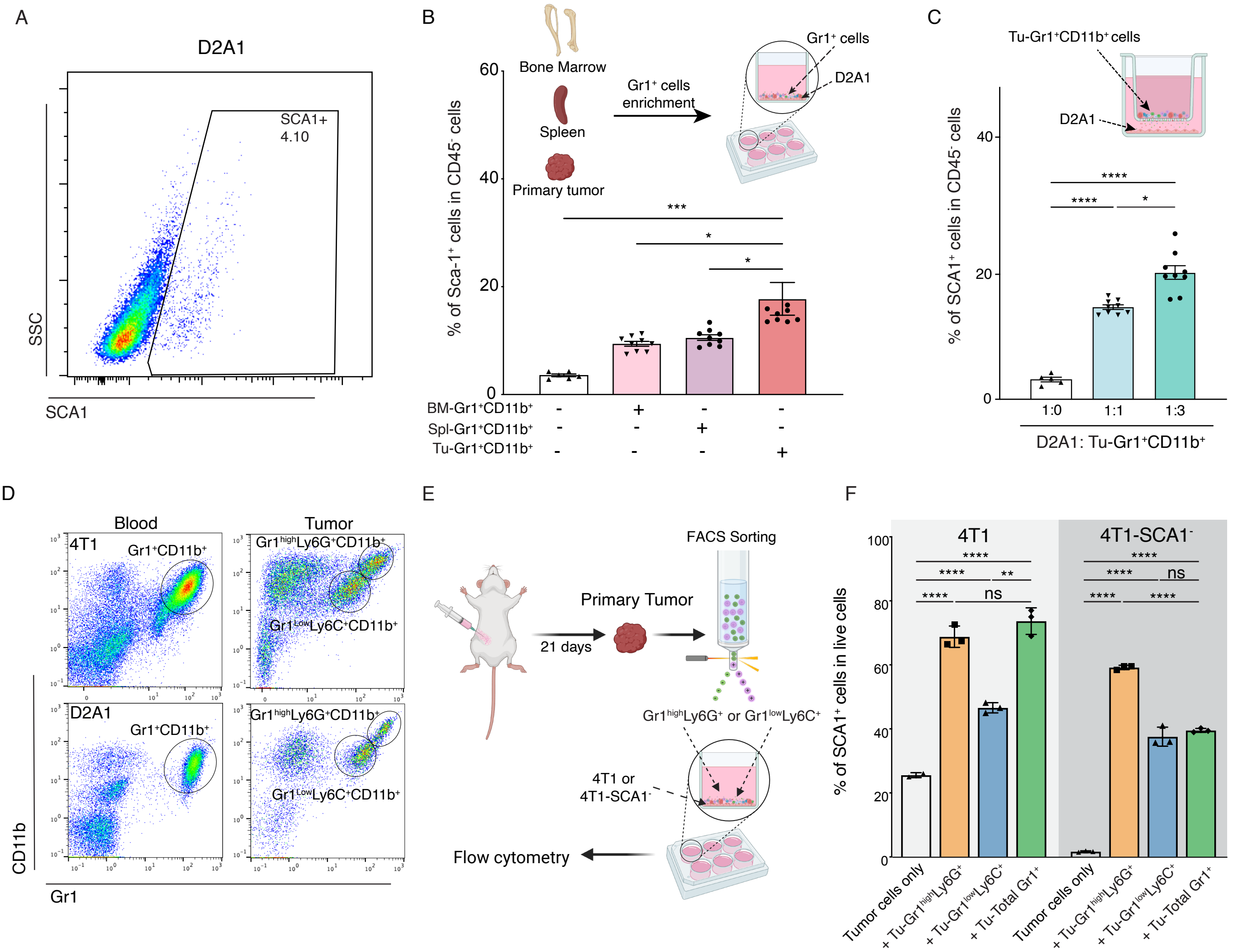

Supplemental Figure 3

Supplemental Figure 3. Tu-Gr1$^+$CD11b$^+$-derived secreted factors promote the enrichment of SCA1$^+$ population

(**A**) Dot plot showing the SCA1$^+$ population in D2A1 tumor cells.

(**B**) MACS-enriched Gr1$^+$ cells from bone marrow, spleen or primary tumor were cocultured with D2A1 tumor cells. After 48 hours, tumor cells were examined for SCA1 expression by flow cytometry. Co-cultures conditions are indicated in the bar graph. (n = 6-9/group).

(**C**) MACS-enriched tumor Gr1$^+$ cells were cocultured with D2A1 tumor cells. After 48 hours, tumor cells were examined for SCA1 expression by flow cytometry. The ratio of tumor cells and Tu-Gr1$^+$CD11b$^+$ is varied from 1:1 to 1:3. (n = 5-9/group).

(**D**) Dot plots presenting Gr1$^+$CD11b$^+$ subpopulations in blood and tumor site of 4T1 (upper panel) and D2A1 (lower panel) tumor-bearing mice.

(**E**) Illustrative scheme showing the experimental design for isolating Gr1^{high} and Gr1^{low} cells from primary tumor 21 days after tumor implantation by FACS sorting. Isolated cells co-cultured for 48 hours with parental 4T1 or sorted 4T1-SCA1$^-$ cells in vitro.

(**F**) Abundance of SCA1 expression in tumor cells was examined by flow cytometry. (n = 3/group).

Data shown as mean ± SEM as representative of 3 independent experiments. *P* values were calculated using one-way ANOVA with Tukey multiple-comparison test.

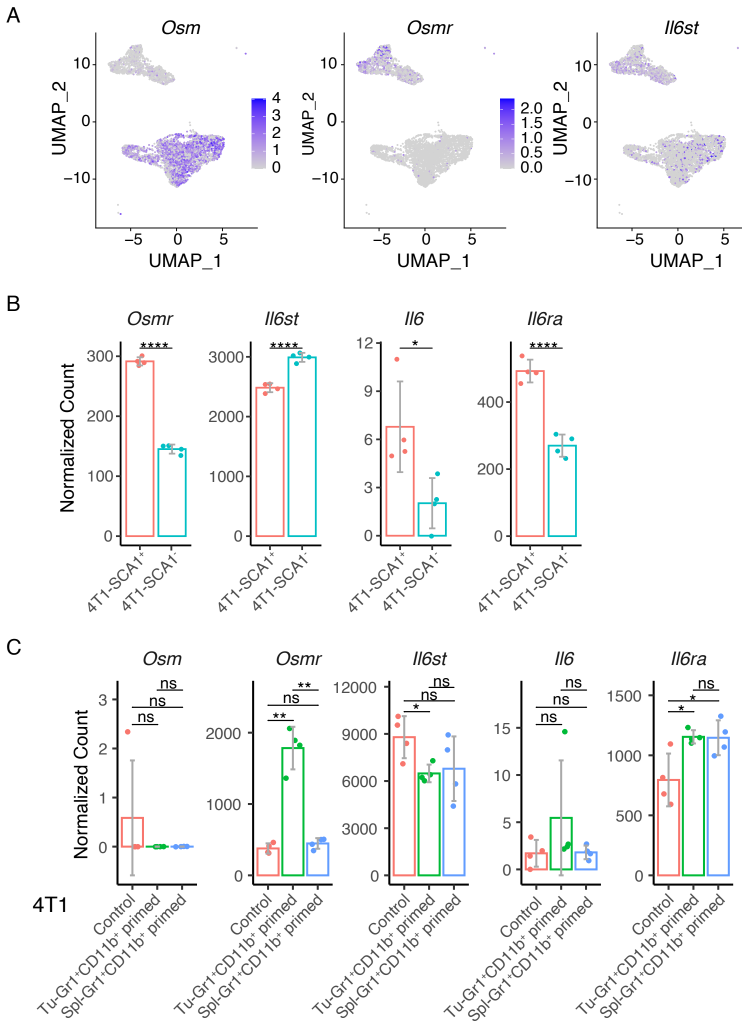

Supplementary Figure 4

Supplemental Figure 4. Transcriptomic analysis of SCA1$^+$ tumor cells

(**A**) UMAP plot showing the mRNA expression pattern of *Osm*, *Osmr*, *Il6st*, *Il6* and *Il6ra* in different cell populations in the Sebastian dataset.

(**B** and **C**) mRNA expression of *Osm*, *Osmr*, *Il6st*, *Il6* and *Il6ra* in 4T1-SCA1$^+$ vs SCA1$^-$ (**B**) and Tu-Gr1$^+$CD11b$^+$ vs Spl$^-$Gr1$^+$CD11b$^+$-primed vs controls 4T1 cells (**C**), based on RNAseq data used to generate the heatmaps shown in Figure 3, A and B, respectively. *Osm* expression is not detected in sorted 4T1 cells. Data are presented as normalized count number ± SD. *P* values were calculated with unpaired two-tailed student's t test for **B**, and unpaired two-tailed student's t test with Holm correction for **C**.

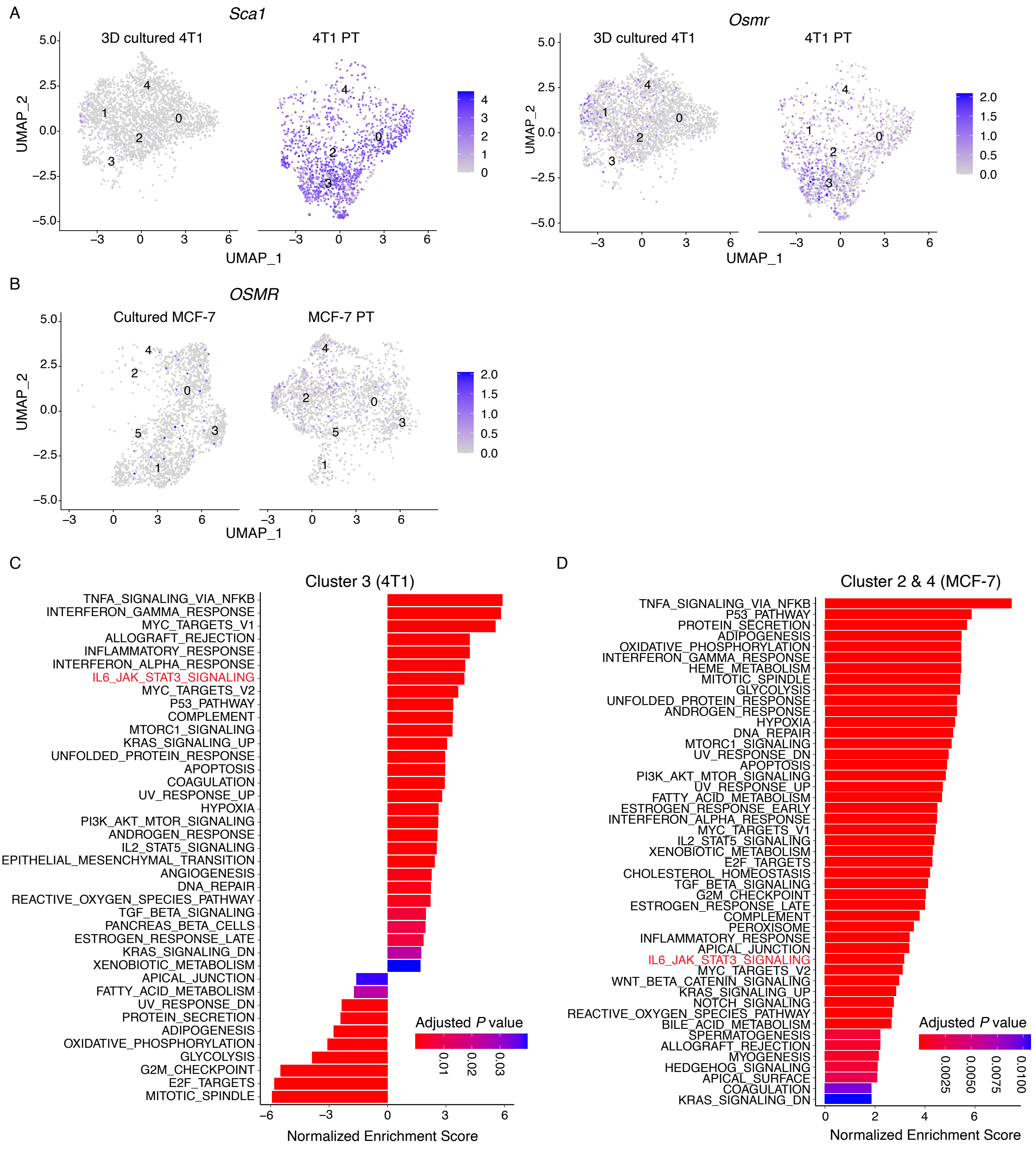

Supplemental Figure 5

Supplemental Figure 5. TME expands the SCA1$^+$ population during in vivo tumor progression

(**A**) UMAP plots showing the expression of *Sca1* and *Osmr* in 4T1 tumor cells in 3D culture or primary tumors (PT). Analysis based on publicly available data (GSM4812003 and GSM3502134).

(**B**) UMAP plot showing the expression of *OSMR* in MCF-7 tumor cells in culture or primary tumors (PT). Analysis based on publicly available data (GSM4681765 and GSM5904917).

(**C-D**) GESA analysis of Hallmark gene sets of cluster 3 in 4T1 data (**C**) and clusters 2 and 4 in MCF-7 data (**D**). Only the signatures with adjusted *P* value < 0.05 are shown. Analyses are based on publicly available data (4T1: GSM4812003 and GSM3502134; MCF-7: GSM4681765 and GSM5904917).

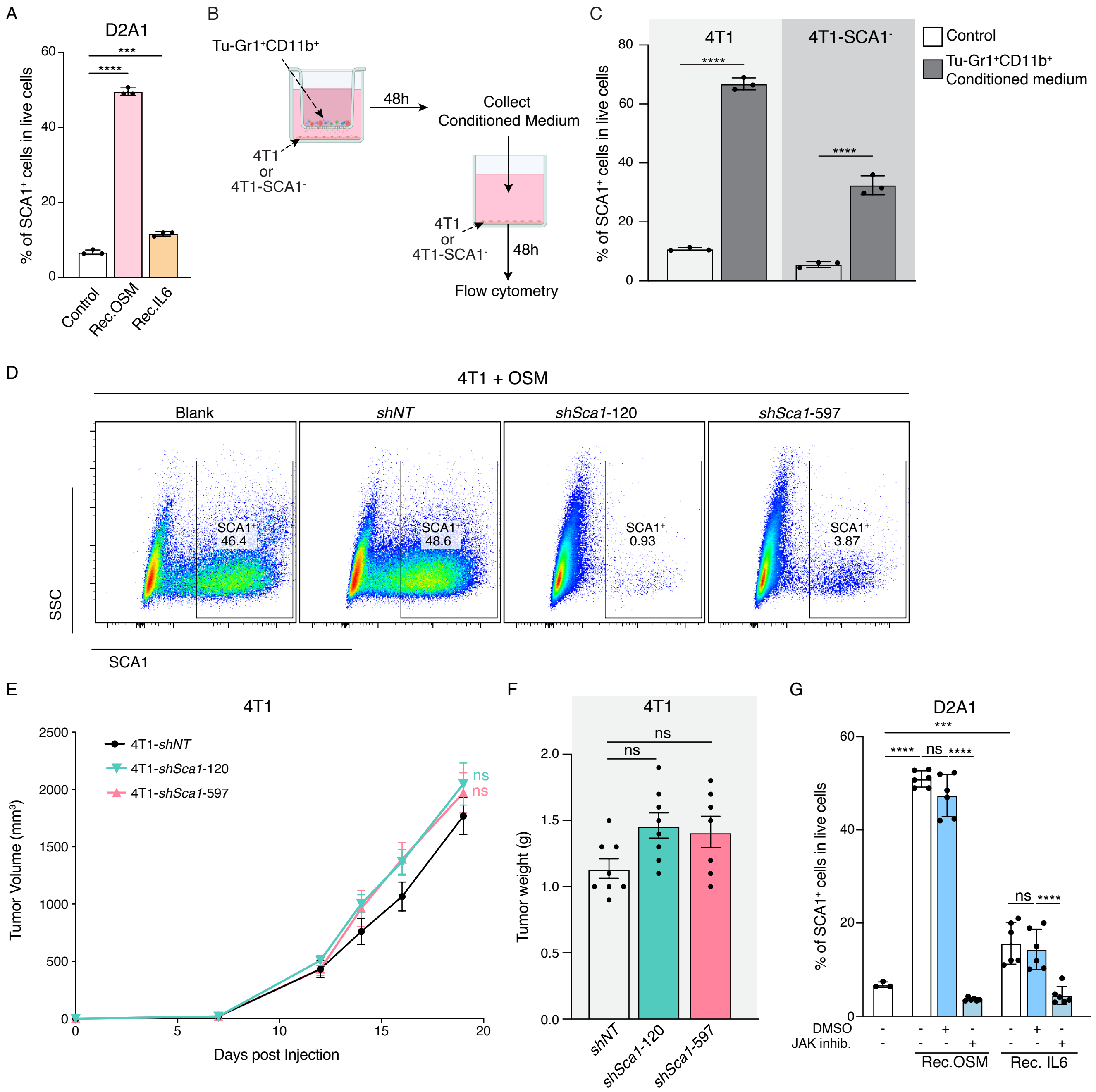

Supplemental Figure 6

Supplemental Figure 6. SCA1^{+} population is modulated by the OSM/IL6-JAK pathway
(**A**) Fraction of SCA1^{+} cells upon exposure of D2A1 cells to recombinant IL6 or OSM protein (10 ng/ml for 48 hours) as determined by flow cytometry. (n=3/group).
(**B** and **C**) Abundance of the SCA1^{+} population in tumor cells cultured for 48 hours in the presence of medium collected from 4T1/Tu-Gr1^{+}CD11b^{+} cocultures (48 hours). SCA1 expression on 4T1 or purified 4T1-SCA1^{-} cells was determined by flow cytometry. (n=3/group).
(**D**) Dot plots presenting *Sca1* KD validation in 4T1 cells treated with recombinant OSM (10 ng/ml) for 48 hours. SCA1 expression was assessed by flow cytometry to show the efficiency of lentivirus.
(**E**) Primary tumor growth for mice injected by 4T1 with *ShNT*, 4T1 transduced with 2 *Sca1* silencing lentivirus (120 and 597). (n=8/group).
(**F**) Tumor weight of mice injected by 4T1-*ShNT*, 4T1-*ShSca1*-120 or *ShSca1*-597 21 days post injection. (n=8/group).
(**G**)Treatment with the JAK inhibitor ruxolitinib (5 μM) of cultured D2A1 cells stimulated with recombinant IL6 or OSM protein (10 ng/ml, 48 hours exposure) as indicated. (n=6/group).
Data shown as mean ± SEM as representative of 3 independent experiments. *P* values were calculated by one-way ANOVA with Dunnett's multiple-comparison test.

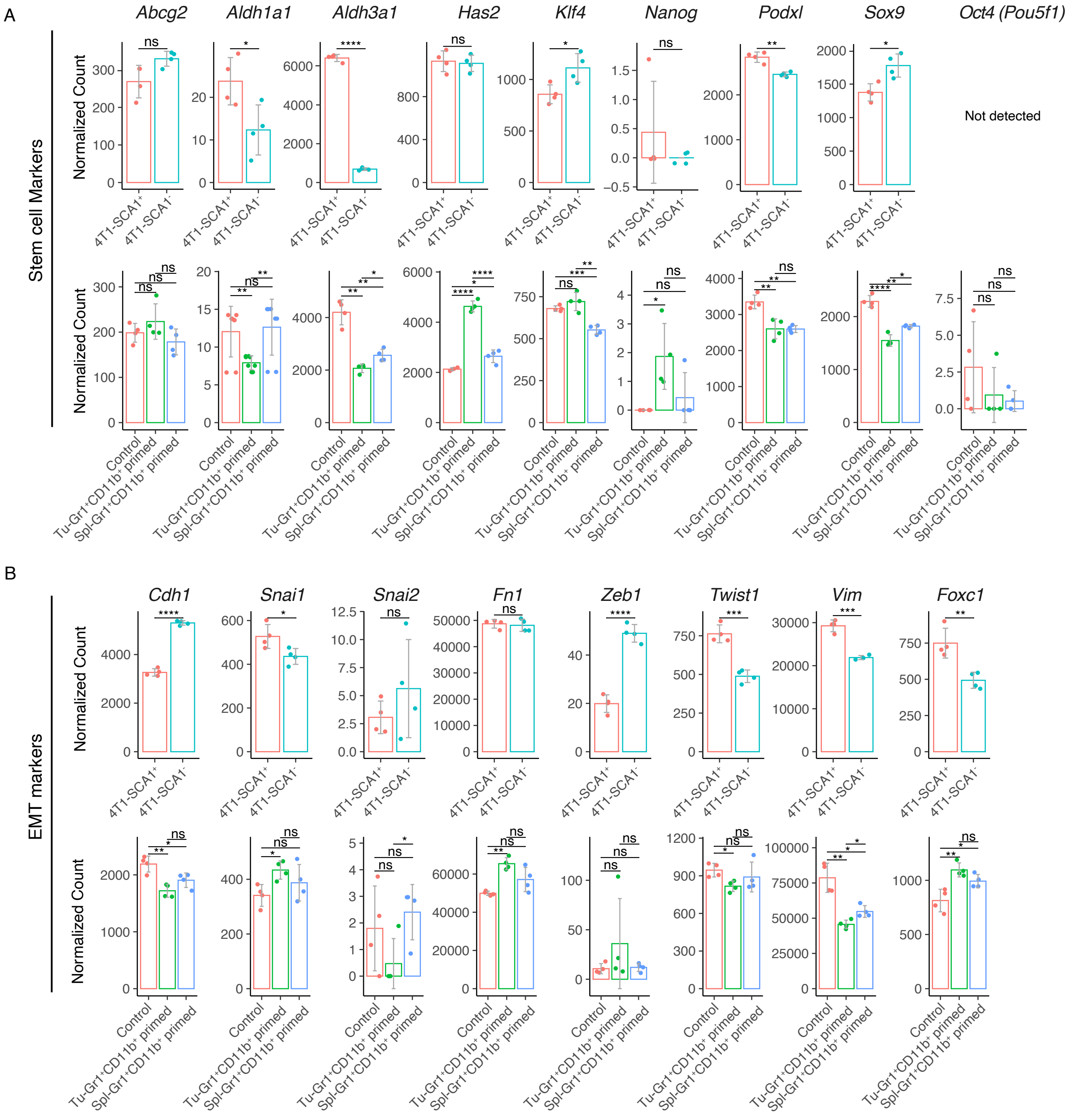

Supplemental Figure 7

Supplemental Figure 7. Expression of selected stem cell/CSC and EMT genes in 4T1-SCA1^{+}, 4T1-SCA1^{-} and differently primed 4T1 cells

Expression of stem cell markers (**A**) and EMT markers (**B**) in 4T1-SCA1^{+} cells, 4T1-SCA1^{-} cells, and parental, Tu-Gr1^{+}CD11b^{+}-educated, Spl-Gr1^{+}CD11b^{+}-educated 4T1 cells, as indicated. Data represent mean values of RPKM ± SD. *P* values were calculated using unpaired two-tailed student's t test with Holm correction.

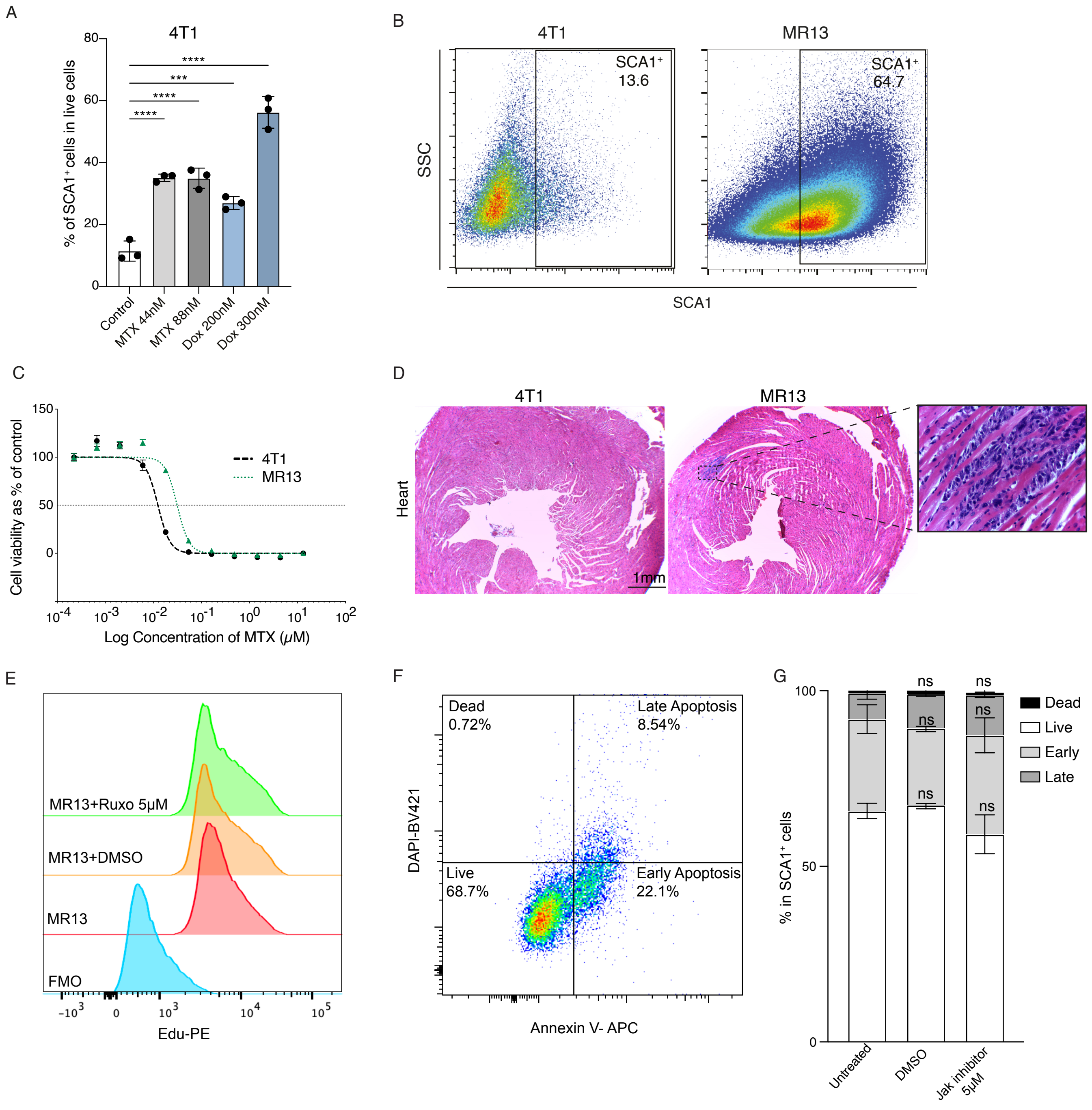

Supplemental Figure 8

Supplemental Figure 8. SCA1$^+$ enriched 4T1 cells and MR13 cells are more resistant to chemotherapy drugs

(**A**) Fraction of 4T1-SCA1$^+$ cells upon short-term (48 hours) methotrexate (MTX, 44 and 88 nM) and doxorubicin (Dox, 200 and 300 nM) treatments. (n=3/group).

(**B**) Dot plots representing SCA1 expression vs SSC determined by flow cytometry in MR13 chemotherapy resistant cells vs parental 4T1 tumor cells in vitro.

(**C**) Dose dependent effect of 48 hours treatment with MTX on 4T1 and MR13 cells' viability. IC50 of MTX for 4T1 and MR13 are 12.5 nM and 31.4 nM, respectively.

(**D**) Illustrative H&E stained heart sections of BALB/c mice 22 days post orthotopic injection with 4T1 cells (left) or MR13 cells (right and insert). (n=8-9/group).

(**E**) Illustrative histogram showing EdU positive MR13 cells after JAK inhibitor-ruxolitinib treatment (72 hours).

(**F** and **G**) Apoptosis rate of MR13 treated with ruxolitinib determined by Annexin V apoptosis test. Live, early apoptotic, late apoptotic and dead cells were defined as indicated in the dot plot (**F**). Percentage of each population presented in **G**. (n=3/group).

Data shown as mean ± SEM as representative of 3 independent experiments. *P* values were calculated using ordinary one-way ANOVA with Dunnett's multiple comparison test

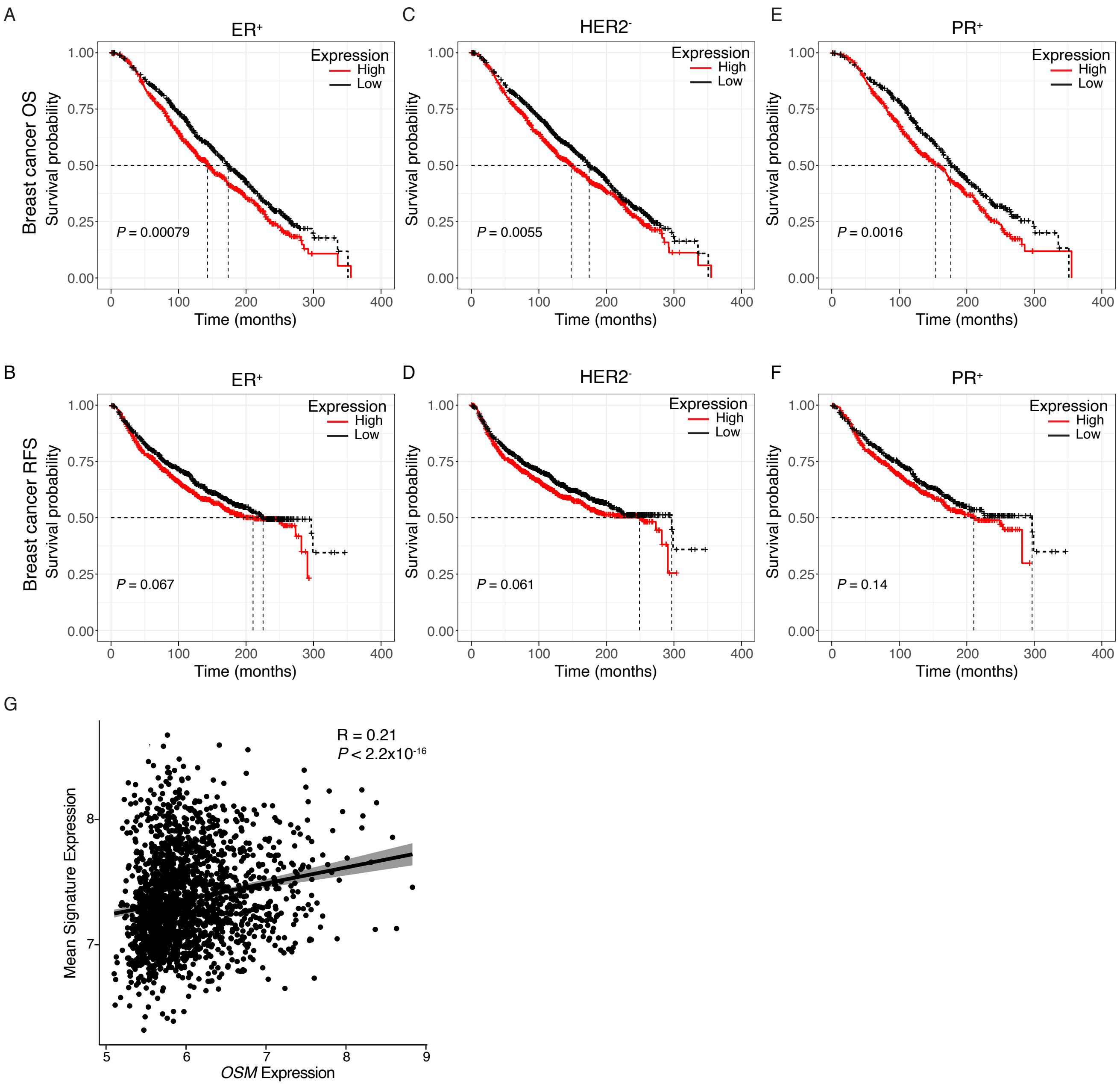

Supplemental Figure 9

Supplemental Figure 9. Orthologues of Tu-Gr1$^+$CD11b$^+$-induced signature and patients' outcome

(**A-F**) Kaplan-Meier curves showing overall survival (OS) (**A**, **C** and **E**) or relapse-free survival (RFS) (**B**, **D** and **F**) for breast cancer patients with ER$^+$ (**A** and **B**), HER2$^-$ (**C** and **D**) and PR$^+$ (**E** and **F**) subtypes according to high or low expression of an orthologue 32 gene signature, based on the Tu-Gr1$^+$CD11b$^+$-induced 4T1 cell signature, in the METABRIC datasets. The *P* values were calculated using the log-rank test and high and low expression levels were stratified by median values.

(**G**) Correlation of the orthologue 32 gene signature, based on the Tu-Gr1$^+$CD11b$^+$-induced signature, with *OSM* expression in the METABRIC datasets. Spearman's correlation coefficient and *P* value are shown.

Supplemental Table 1. Gene signatures related to Figure 3

Supplemental Table 2. CellPhoneDB analysis results

Supplemental Table 3. Tu-Gr1^{+}CD11b^{+}-induced signatures
